# Global mapping of transcription start sites and promoter motifs in the symbiotic α-proteobacterium *Sinorhizobium meliloti* 1021

**DOI:** 10.1186/1471-2164-14-156

**Published:** 2013-03-07

**Authors:** Jan-Philip Schlüter, Jan Reinkensmeier, Melanie J Barnett, Claus Lang, Elizaveta Krol, Robert Giegerich, Sharon R Long, Anke Becker

**Affiliations:** 1 Institute of Biology III, Faculty of Biology, Albert-Ludwigs-Universität Freiburg, Freiburg, Germany; 2 LOEWE Center for Synthetic Microbiology (SYNMIKRO) and Department of Biology, Philipps-Universität Marburg, Marburg, Germany; 3 Center for Biotechnology (CeBiTec), Bielefeld University, Bielefeld, Germany; 4 Department of Biology, Stanford University, Stanford, CA, 94305, USA

**Keywords:** Transcription, RNAseq, Transcription start site, Promoter, Sigma factor, *Sinorhizobium meliloti*, mRNA, sRNA, Antisense RNA

## Abstract

**Background:**

*Sinorhizobium meliloti* is a soil-dwelling α-proteobacterium that possesses a large, tripartite genome and engages in a nitrogen fixing symbiosis with its plant hosts. Although much is known about this important model organism, global characterization of genetic regulatory circuits has been hampered by a lack of information about transcription and promoters.

**Results:**

Using an RNAseq approach and RNA populations representing 16 different growth and stress conditions, we comprehensively mapped *S. meliloti* transcription start sites (TSS). Our work identified 17,001 TSS that we grouped into six categories based on the genomic context of their transcripts: mRNA (4,430 TSS assigned to 2,657 protein-coding genes), leaderless mRNAs (171), putative mRNAs (425), internal sense transcripts (7,650), antisense RNA (3,720), and *trans*-encoded sRNAs (605). We used this TSS information to identify transcription factor binding sites and putative promoter sequences recognized by seven of the 15 known *S. meliloti* σ factors σ^70^, σ^54^, σ^H1^, σ^H2^, σ^E1^, σ^E2^, and σ^E9^). Altogether, we predicted 2,770 new promoter sequences, including 1,302 located upstream of protein coding genes and 722 located upstream of antisense RNA or *trans*-encoded sRNA genes. To validate promoter predictions for targets of the general stress response σ factor, RpoE2 (σ^E2^), we identified *rpoE2*-dependent genes using microarrays and confirmed TSS for a subset of these by 5^′^ RACE mapping.

**Conclusions:**

By identifying TSS and promoters on a global scale, our work provides a firm foundation for the continued study of *S. meliloti* gene expression with relation to gene organization, σ factors and other transcription factors, and regulatory RNAs.

## Background

Transcription is the first committed step of gene expression in prokaryotes, and as such is highly regulated. Promoter sequences direct the transcription of both coding and non-coding RNAs by acting as target sites for specific RNA polymerase binding and activity [1-3]. Bacteria employ an ingenious machinery to adapt effectively and economically to conditions of stress or environmental changes. The initial mechanism of transcription regulation is based on recruitment of certain sigma (σ) factors by RNA polymerase core enzyme (RNAP). Complex formation with σ factors is essential for RNAP binding to a particular promoter sequence and thus for transcription initiation [3].

 Genome-wide identification of promoter sequences facilitates identification of DNA-binding sites for regulatory proteins and also provides insights into the organization of transcriptional units. RNAseq approaches have allowed the large-scale identification of transcription start sites (TSS) in the ε-proteobacterium *Helicobacter pylori* and the cyanobacteria *Synechocystis* sp.*, Synechococcus elongatus,* and *Anabaena* sp., facilitating the characterization of promoter sequence motifs upstream of the TSS. These approaches revealed an unexpected abundance of *cis*-encoded antisense RNAs (asRNA) and *trans*-encoded sRNAs (sRNA), and thereby illuminated a previously unknown dimension of prokaryotic transcriptional activity [4-7]. This picture is consistent with RNAseq studies in diverse Gram-positive and Gram-negative bacteria [8-17].

 The α-class of the Proteobacteria comprises diverse bacteria with complex lifestyles, including obligate and facultative plant- and animal-associated bacteria (engaging in both mutualistic and pathogenic interactions), phototrophs, chemoorganotrophs, and chemolithotrophs [18]. In addition, eukaryotic mitochondria are proposed to have arisen from an ancient α-proteobacterium [18]. Despite their importance, little is known about α-proteobacterial transcriptome organization. This study presents the first comprehensive mapping of TSS and assignment of identified promoter sequences to σ factors in an α-proteobacterium. *Sinorhizobium meliloti* exists either in a free-living life-style in the soil or in symbiosis with a leguminous plant host. In the symbiotic relationship, the bacteria inhabit root nodules, differentiate into polyploid bacteroids, and fix nitrogen to the benefit of the host [19]. Functions for these distinct lifestyles are encoded in the tripartite *S. meliloti* genome: a single chromosome (3.54 Mbp) and two megaplasmids, pSymA (1.35 Mbp) and pSymB (1.68 Mbp) [20-22].

 To adapt to environmental changes or stress situations, *S. meliloti* can draw on a set of 15 σ factors [20,23]. RpoD (σ^70^) provides for housekeeping functions, while alternative σ factors are usually involved in adaptation to specific stresses or growth conditions [24]. RpoN (σ^54^) is essential for transcription of nitrogen fixation-related genes [25]. Two RpoH σ factors, with sequence similarity to the *Escherichia coli* heat shock σ^32^, were identified in *S. meliloti*[26]; RpoH1 (σ^H1^) was found to be largely responsible for adaptation to heat shock, oxidative stress, and pH changes, whereas the role of RpoH2 (σ^H2^) is largely unknown [26]. At least 11 σ factor genes (*rpoE1*-*rpoE10*, *fecI*) are annotated in the *S. meliloti* genome as encoding extracytoplasmic function (ECF) σ factors, which are usually regulated by anti-σ factors. The ECF σ factor, RpoE2 (σ^E2^), was characterized as the regulator of at least 44 genes, including *rpoH2* and *rpoE5,* and was inferred to be a global regulator of general stress adaptation and the hyperosmotic stress response [27,28].

 The RNAseq approach reported in this study obtained 17,001 experimentally mapped TSS, including both protein-coding and non-coding transcripts. We were able to predict 2,847 σ factor-specific promoter sequences in appropriate distance to a TSS. This newly defined landscape of TSS and promoter motifs increases our knowledge of promoter properties and will facilitate further analyses of transcriptional and post-transcriptional regulation processes in *S. meliloti*.

## Results and discussion

### Global mapping of transcription start sites

To comprehensively map TSS of the *S. meliloti* 1021 transcriptome, RNA populations derived from 16 different growth and stress conditions were pooled and used for cDNA library preparation (Methods). These conditions included exponential and stationary phase growth in three different media, temperature and pH shifts, oxidative and high salt stress, microoxia, carbon or nitrogen starvation, and exposure to the plant-secreted flavonoid, luteolin. This flavonoid induces expression of bacterial nodulation genes, which are required for the establishment of symbiosis [29]. *S. meliloti* 1021 carries a mutation in *expR* encoding a global LuxR-type transcriptional regulator engaged in quorum sensing. This regulator affects multiple processes including exopolysaccharide biosynthesis and motility [30-32]. In order to map ExpR-dependent transcripts, which would otherwise be lacking, RNA from an *expR*^+^ derivative of *S. meliloti* 1021 was also included in our pool. Enzymatic treatment of RNA samples with terminator 5^′^-phosphate-dependent exonuclease resulted in enrichment of transcripts with primary 5^′^ ends, prior to cDNA synthesis and deep sequencing (Methods).

 Our procedure for mapping and classifying TSS is diagrammed in Additional file 1: Figure S1 and described in detail in Methods. Briefly, 37,792,608 reads were obtained, of which 11,230,166 passed all filtering criteria, and subsequently were subjected to TSS determination and classification. TSS assigned to tRNAs, repeats, and transposons are listed separately (Additional file 2: Table S1).

**Figure 1:**
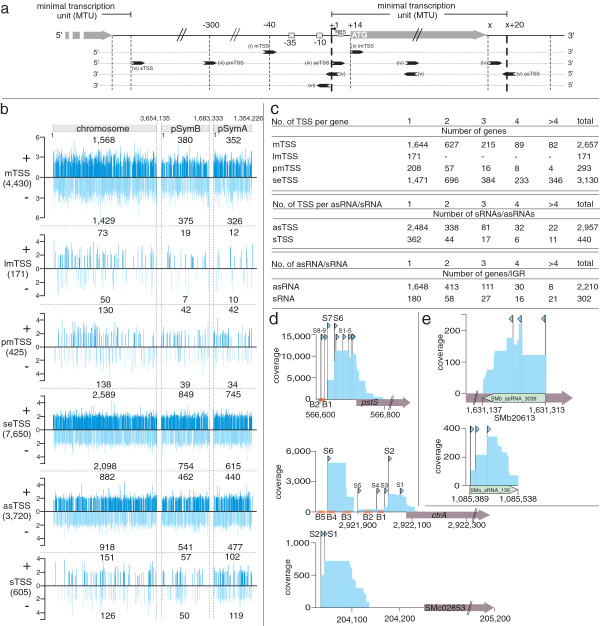
**Identification of transcription start sites (TSS) in the *S. meliloti *genome.** (**a**) Schematic showing parameters of TSS types based on the minimal transcription unit model described in Methods. **(i)** TSS of an mRNA. **(ii)** TSS of a leaderless transcript. **(iii)** TSS of a putative mRNA. **(iv)** TSS of a sense transcript. **(v)** TSS of a *cis*-encoded antisense RNA. **(vi)** TSS of a *trans*-encoded sRNA. (**b**) Strand and replicon specific distribution of TSS by category. The y-axis indicates log_10_ coverage for mapped reads. (**c**) Number of protein-coding genes, asRNAs and sRNAs possessing 1, 2, 3, 4 or >4 TSS. The last two rows give the number of genes or IGRs possessing 1, 2, 3, 4 or >4 asRNAs or sRNAs. (**d**) TSS associated with *pstS*, *ctrA,* and SMc02853 (indicated by blue triangles). Putative transcription factor binding motifs are shown as orange rectangles. **(e)** TSS associated with SMb_asRNA_3039 and SMa_sRNA_136 (indicated by blue triangles).

Overall, 17,001 TSS, not including those listed in Table S1, were grouped into six categories based on their genomic context with respect to a minimal transcription unit (MTU) model (Methods; Figure 1a; Additional file 2: Table S2). TSS representing the prominent 5^′^ end of a sequence contig overlapping in sense orientation a region of 54 nt upstream of the start codon of protein-coding genes were classified as (i) TSS of mRNAs (mTSS). 54 nt were defined as the minimal region upstream of the ATG to cover promoter motifs and the ribosome binding site, which are more likely to be associated with an mRNA than to a *trans*-encoded sRNA. (ii) TSS were assigned to leaderless transcripts (lmTSS), if matching the first nucleotide of the translation initiation codon. The class of (iii) putative TSS of mRNAs (pmTSS) comprises TSS that are difficult to distinguish: for any given pmTSS, it is uncertain if the TSS represents an mRNA of a protein-coding gene possessing a long 5^′^-UTR or a TSS of a *trans*-encoded sRNA. (iv) Sense TSS (seTSS) represent internal transcripts in the same orientation as, and located within, protein-coding genes. (v) TSS of *cis*-encoded antisense RNAs (asTSS) are orientated in antisense to protein-coding target genes, and (vi) TSS of *trans*-encoded sRNAs (sTSS) are located in intergenic regions (IGR) and within a defined distance from neighboring genes. Figures 1b and 1c give an overview of the location and number of TSS in each category, which are discussed in more detail below.

### TSS categories

#### *mTSS* (TSS associated with mRNAs)

**Figure 2:**
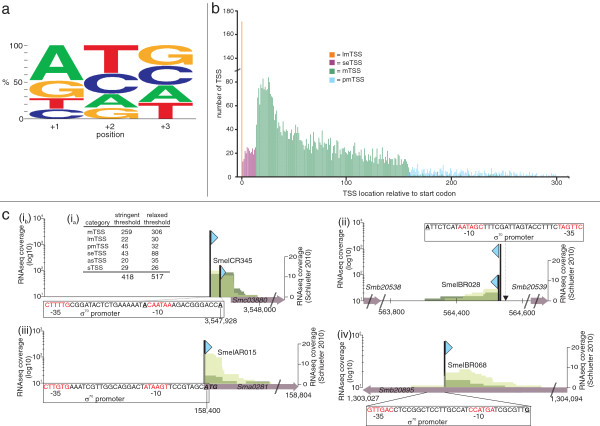
**Characteristics of *S. meliloti *transcripts.** (**a**) Nucleotide composition for the first 3 nt of mRNAs Positions +1, +2, and +3 are indicated, of which +1 is the TSS. (**b**) Distance of TSS to start codons. In **orange**: TSS of leaderless transcripts (lmTSS); **purple**: TSS located close to the start codon that were classified as putative TSS of sense sRNAs (seTSS); **green**: TSS of mRNAs, including *cis*-encoded mRNA leader sequences of [17]**blue**: TSS of putative mRNAs (pmTSS). **(c)** (i_a_) Transcription units identified by both the TSS-specific RNAseq and the small RNA-specific RNAseq approach (applying stringent [17] and relaxed thresholds). (i_b_) mRNA leader sequence (SmelCR345), (ii) sRNA (SmelBR028), (iii) leaderless transcript (SmelAR015) and (iv) asRNA (SmelBR068). TSS are indicated by blue triangles.

A total of 4,430 mTSS were assigned to 2,657 protein-coding genes (Figures 1b and 1c; Additional file 2: Table S2). The total number of *S. meliloti* annotated protein- coding genes is 6296 [33], and 1090 operons were predicted [34]. Assuming a rough estimate of three co-transcribed genes per operon, we have identified TSS for most protein-coding genes in the *S. meliloti* genome. About 60% (1,644 genes) appear to contain a single TSS, while the remaining genes contain multiple TSS. At least 12 putative TSS were identified for each of the genes SMc01904, SMc02122, and SMc02396 (Additional file 2: Table S2). The most frequent initiating nucleotide for mTSS transcripts was A (47.9%), followed by G (23.6%), T (14.8%), and C (13.7%) (Figure 2a). This agrees with observations that purine nucleotides are the most common initiating nucleotide in bacteria [15,35], a preference that may be explained by the relatively larger pool size of purine vs. pyrimidine nucleotides [36].

The existence of alternative promoters for a single gene is one explanation that could account for multiple TSS, as exemplified by *pstS,* which encodes a phosphate-binding periplasmic protein, and was previously shown to have two TSS located downstream from two PhoB binding motifs (B1 and B2) corresponding to TSS S8 and S9 of Figure 1d [37]. Similarly, *ctrA*, which encodes a global cell cycle regulator, showed two strongly accumulating transcripts corresponding to TSS S2 and S6 (Figure 1d) that were previously identified downstream from conserved promoter motifs (Figure 1d) [38]. Alternatively, post-transcriptional processing of the 5^′^-UTR of mRNAs may account for multiple mTSS located downstream of TSS associated with conserved promoter motifs, as is plausible for six TSS of *pstS* (S1, S3, S4, S5, S8, and S9 in Figure 1d) and four TSS of *ctrA* (S1, S3, S4, and S5 in Figure 1d).

The average mTSS-to-start-codon distance was ~ 68 nt (Figure 2b), somewhat longer than previously reported for *Salmonella enterica* serovar *Typhimurium*[15] and *H. pylori*[4]. Moreover, the average distance increases to ~80 nt if pmTSS lengths (see below) are included in the calculation. In 40% of all cases, the distance between the mTSS and start codon is between 40 and 100 nt. The maximum distance was 309 nt for the mRNA leader transcript of SMc02724 (Additional file 2: Table S2; [17]). 5^′^-UTRs longer than 100 nt were found preceding 1,041 genes (1,466 if pmTSS are also included). This indicates that long 5^′^-UTRs are not unusual in *S. meliloti* and may even signify complex mechanisms of gene regulation, as is likely the case for *ctrA* (Figure 1d; [38]).

 To identify new protein-coding genes that were missed in previous genome annotations [20,33], we followed the procedure described in Methods and outlined in Additional file 1: Figure S2. This included screening for translational signals, such as ribosome binding sites (RBS) and translational start and stop codons. This approach yielded 221 TSS, associated with 71 new and 150 alternative translational units (Additional file 2: Table S3). To increase the probability of annotating only genuine open reading frames (ORFs), we searched the NCBI nonredundant protein database for orthologs of these newly identified genes. Genes that had orthologs in other α-proteobacteria were named according to the scheme, *SMx_ORF_x,* and added to the GenDB *S. meliloti* genome database [39].

#### *lmTSS* (TSS associated with leaderless transcripts)

171 TSS of leaderless transcripts mapped to the first nucleotide of the corresponding start codon (Figures 1a, 1b, and 2b). Only a single TSS was found for 112 of these lmTSS, with the remainder found in combination with mTSS (n=52), pmTSS (n=4), or both (n=3) (Additional file 2: Table S2). Leaderless transcripts were also observed in *Synechocystis* sp. PCC6803, *E. coli*, *Rhodobacter sphaeroides*, and *Pseudomonas putida*[40-43]. All lmTSS initiate with an AUG triplet, consistent with results in *E. coli* showing that the 5^′^-terminal AUG of leaderless mRNAs is important for ribosome recognition and interaction [44], and that non-AUG start codons are inefficient [45]. Also, a recent study proposed a mechanism for leaderless mRNA translation in *E. coli*[40]. In this mechanism, the *mazEF* stress-induced toxin-antitoxin system, in particular the endoribonuclease MazF, plays an important role in maturation of both leaderless mRNAs and a novel, processed 16S rRNA. Assembly of the ribosome with this novel rRNA molecule results in an alternative translational machinery, which is able to translate two classes of leaderless transcripts: those processed via MazF and naturally occurring leaderless mRNAs [40]. In *S. meliloti*, our observation that most genes with leaderless transcripts possess a single TSS (n=112), suggests that naturally occurring leaderless mRNAs are preferred over processed leaderless transcripts. It is possible that the 59 leaderless mRNAs for which an alternative TSS was identified were processed via endoribonucleases similar to MazF in *E. coli*, but such a mechanism has not yet been described in *S. meliloti*.

Organization of protein-coding genes into operons is a common feature in bacterial genomes. Recently, we reported 67 experimentally validated operons and 1,090 predicted operons in the *S. meliloti* 1021 genome [34]. TSS for 47 of those experimentally validated operons and 613 of the predicted operons were identified in our RNAseq data (Additional file 2: Table S4).

#### *pmTSS* (TSS associated with putative mRNAs)

A special situation is illustrated by 425 pmTSS, of which 122 represent the only TSS identified for the corresponding gene (Figure 1b; Additional file 2: Table S2). In many cases, it is unclear if these pmTSS represent mRNAs with unusually long 5^′^-UTRs or non-coding transcripts. One such example is illustrated by SMc02853, which possesses two pmTSS (Figure 1d). On the other hand, the most distal TSS of *ctrA* (S6 in Figure 1d) almost certainly represents a transcript with a long 5^′^-UTR (291 nt), because CtrA was shown to bind upstream of this TSS [38]. CtrA also binds to four additional sites downstream of the S6 TSS suggesting a complex mechanism of autoregulation (Figure 1d; [38]).

#### *seTSS* (TSS associated with internal sense transcripts of ORFs)

Sense TSS must meet two criteria: they must be located in the same orientation as a protein-coding gene and within the corresponding ORF. Sense transcripts are the largest group of TSS in our study: 7,650 were classified as belonging to sense transcripts; and therefore, approximately half of all identified TSS correspond to internal fragments of mRNA transcripts (Figure 1c; Additional file 2: Table S2).

A high proportion of sense transcripts was also observed in RNAseq studies of other prokaryotes, e.g. *Synechocystis* sp. PCC6803 and *Anabaena* sp. PCC7120, but the functional role of these RNA fragments, if any, remains to be elucidated [5,6]. It is possible that sense transcripts may act as target mimicry molecules that sequester sRNAs, asRNAs, or ribonucleases from their respective mRNA target domains [5]. Presumably, a small proportion of sense fragments may represent alternative mRNAs that allow for synthesis of shorter protein isoforms in *S. meliloti*. However, a more likely explanation is that despite the procedure we used to enrich for transcripts with primary 5^′^ ends, the majority of sense fragments correspond to abundant processed derivatives of their respective mRNAs rather than independent, primary mRNAs transcribed from their own promoters [17].

#### *asTSS* (TSS of *cis*-encoded antisense sRNAs)

*Cis*-encoded antisense RNAs act as regulatory RNA via perfect binding to their corresponding target mRNAs encoded on the opposite DNA strand. The first genome-wide RNAseq-based screen for non-coding transcripts in * S. meliloti* identified 117 asRNAs [17]. However, antisense RNA detection was limited in that study, because the protocol was designed to preferentially detect short RNAs. This study greatly increased the number of antisense transcripts with the identification of 3,720 asTSS that were assigned to 2957 asRNAs associated with the noncoding strand of protein-coding genes (Figures 1b and 1c; Additional file 2: Table S2). Approximately 35% of the protein-coding genes in *S. meliloti* (2,210 out of 6,296 genes) had antisense transcripts (Figure 1c; Additional file 2: Table S2). For 1,648 target genes, a single asTSS was identified, while the remaining genes appear to contain two or more asTSS (Figure 1c). An example of an antisense RNA with three TSS, SMb_asRNA_3039, is shown in Figure 1e. Occurrence of numerous antisense transcripts associated with a single protein-coding gene most likely represents processed products of a single, primary non-coding asRNA, rather than transcripts of independent asRNA genes. Examples are SMb21548, SMc02498, and SMc03761 with 50, 14, and 12 asTSS, respectively (Additional file 2: Table S2). The three replicons, chromosome, pSymA, and pSymB represent 54.6%, 20.2%, and 25.2% of the total genome size in bp. Taking into account the different replicon sizes, antisense transcripts were weakly overrepresented on pSymA (23.9%) and pSymB (26.5%) (Figure 1b; Additional file 2: Table S2). The strand-specific location of antisense transcripts on pSymB shows a slight preference for the minus strand, whereas it is virtually equal on the remaining replicons (Figure 1b).

 The observed features for *cis*-encoded antisense sRNAs in *S. meliloti* are in good agreement with other bacterial transcriptomes. Three studies detected antisense transcription of ~27% to ~50% of the annotated protein-coding genes in two cyanobacteria and *H. pylori*[4-6]. It is tempting to speculate that antisense transcriptional activity is widespread in prokaryotic genomes. However, antisense transcriptional activity represented less than 5% of the protein-coding genes in *Chlamydia trachomatis*, *Staphylococcus aureus*, *P. syringae*, *Vibrio cholerae, and S. enterica*[12,15,46-48]. It is unknown whether this variation reflects differences in experimental and data analysis procedures or biological mechanisms.

 The total length of each transcript in our study is unknown due to the method we used for cDNA library preparation. Prokaryotic asRNAs are generally short transcripts of approximately 100 to 300 nt [2]. This number is in good agreement to the size range (59 to 258 nt) of 117 *S. meliloti* asRNAs identified in Schlüter et al. [17]. However, several asRNAs of other bacterial species were as long as 7,000 nt [49-53].

Depending on the location of an asRNA relative to its target gene, various mechanisms have been proposed. In * S. meliloti,* 441 predicted asRNAs overlapped the 5^′^-UTR of the associated mRNA (Additional file 2: Table S2). These may function as described for SymR in *E. coli* and RnaG in *Shigella flexneri:cis*-encoded asRNAs, antisense to 5^′^-UTR domains of their relative target genes, are able to modulate transcription, translation efficiency, and mRNA stability [54,55]. In *S. meliloti,* 250 asRNAs overlap the 3^′^-UTR of their particular target genes (Additional file 2: Table S2) and therefore, might influence the target mRNA stability in a similar fashion as described for the RatA/TxpA toxin-antitoxin system in *B. subtilis*. Duplex formation by *ratA* and *txpA* transcripts leads to *txpA* mRNA degradation, and thus prevents toxin synthesis [56,57]. The largest group of *cis*-encoded asRNAs in *S. meliloti* (n=2,266) comprises transcripts antisense to the protein-coding domain of their target mRNA (Additional file 2: Table S2). Several regulatory mechanisms have been postulated for this type of asRNA. An example of targeted co-degradation of an mRNA/asRNA duplex is illustrated by *isiA*/IsrR of *Synechocystis* sp. PCC6803 [58,59]. Other regulatory mechanisms do not focus on the asRNAs themselves, but on the strength of their respective promoters, RNAP elongating rates, and RNAP competition events. Three mechanisms of transcriptional interference, promoter collision, promoter occlusion, and “sitting duck” interference (the dislodgement of RNAP slowly initiating transcription from one promoter by an elongating RNAP from another promoter), were proposed to explain the interfering effects of RNAP complexes acting in opposite directions [60].

#### *sTSS* (TSS of *trans*-encoded sRNAs)

* Trans*-encoded sRNAs are usually located in IGRs, and in contrast to antisense transcripts, do not overlap their target genes. In total, 605 sTSS were associated with *trans*-encoded sRNAs (Figure 1b; Additional file 2: Table S2). As described for SmelA075, SmelA060, and SmelA072 in *S. meliloti,* and GadY in *E. coli*, processed variants of non-coding transcripts are not unusual in bacteria [17,61]. Thus, we assigned closely neighboring TSS to a single sRNA region. An example for newly identified SMa_sRNA_136 is shown in Figure 1e. The majority of *trans*-encoded sRNAs (n=362) were assigned only a single TSS, while 78 exhibited at least one alternative TSS (Figure 1c; Additional file 2: Table S2). Therefore, our RNAseq approach identified 440 sRNAs located in 302 IGRs (Figure 1c; Additional file 2: Table S2). Newly identifed sRNAs were named following the scheme SMx_sRNA_x and added to the *S. meliloti* GenDB database [39].

 Comparison of our RNAseq approach to that of Schlüter et al. [17], confirmed 259 mTSS, 29 sTSS, 20 asTSS, and 43 seTSS (Figure 2c; Additional file 2: Table S2). For further validation, we compiled an additional set of transcripts from the previous 454 RNAseq data [17], but applied less stringent criteria (Methods). Comparison of the additional set of transcripts to those of this study showed concordance for 517 (Additional file 2: Table S2). For transcripts identified in both data sets, locations of the 3^′^ ends were retrieved from the previous 454 RNAseq data and are provided in Additional file 2: Table S2. Examples of four such transcripts are shown in Figure 2c.

 Our RNAseq analysis revealed 440 sRNAs; therefore, the ratio of CDS to *trans*-encoded sRNAs in the *S. meliloti* genome is 14.3 (Figure 1c; Additional file 2: Table S2). Somewhat lower CDS/*trans*-encoded sRNA ratios were observed in *V. cholerae, Anabaena* sp. PCC7120, and *Synechocystis* sp. PCC6803, with proportions of 8, 4.4, and 10, respectively [5,6,48]. In *S. meliloti*, the majority of sRNA genes (n=180) are located as single sRNA loci in a single IGR. However, 85 IGRs harbor at least two or three and 37 IGRs harbor at least four sRNA genes (Figure 1c).

 Although pSymA represents only 25.2% of the total genome size in bp, 40.9% of the *trans*-encoded sRNAs identified in this study mapped to pSymA, indicating an over-representation of sRNA genes on pSymA and an under-representation on the chromosome (40.7%) and pSymB (18.4%) (Figure 1b; Additional file 2: Table S2). Considering that many genes required for symbiosis are located on pSymA [21], it is tempting to speculate that *trans*-encoded sRNAs play an important role in post-transcriptional regulation of the symbiotic genes.

Aside from the previously identified 6S RNA (SMc_sRNA_307), the signal recognition particle (SMc_asRNA_791), and the tmRNA (SMc_sRNA_283) [17], assigning functions to sRNAs remains a complex challenge. This study extends our knowledge of sRNAs and 5^′^-UTRs and consequently will facilitate prospective functional analyses of sRNA/5^′^-UTR interactions.

### Comparison to published TSS data

We compared 124 *S. meliloti* 1021 TSS, published over the past several decades, to our RNAseq TSS (Additional file 2: Table S5). This set of previously published TSS is biased toward *rpoH*-dependent genes because 69 of the 124 TSS were reported in a single study [62]. Of the 124 published TSS, 60% matched an RNAseq TSS exactly, 11% differed by only 1–4 nt, and 7% differed by ≥5 nt. The remaining 27 previously reported TSS were not identified in our RNAseq data. Some of these may be explained by the different growth conditions tested in each study. For example, *fixA* and *nifH* are expressed primarily in symbiosis, a condition that was not included in our study for technical reasons. Additionally, Barnett et al. [62] used different growth media than we did, which may explain why we failed to identify 11 *rpoH*-dependent TSS. Two genes, *nodD3* and *syrA*, previously reported to have low expression and extremely long 5^′^-UTR [63,64], were also absent in our RNAseq data. We identified a TSS for *ndvA* in our RNAseq data that was misclassified by our automated system as belonging to the 5^′^-UTR of an asRNA (Additional file 2: Table S5). Other reported TSS, such as those corresponding to *hypM*, *rpoN*, and *trkH*, had matching reads in our RNAseq data, yet failed to meet the coverage cutoff. These examples illustrate the delicate balance between sensitivity and specificity in global studies; in our study, a cutoff of ≥30 reads was chosen to provide the best balance between identification of true positives and background noise.

As previously discussed, a major strength of our study is the comprehensiveness of our data. Depending on the location of the specific primer(s) chosen, alternate TSS may be missed by primer extension or 5^′^ RACE mapping. Hence, in comparing our TSS data to published reports, we identified 45 genes with multiple TSS, where just one TSS had been reported before (Additional file 2: Table S5). In sum, the TSS identified in this study are in good agreement with previous reports.

### Promoter prediction

**Figure 3:**
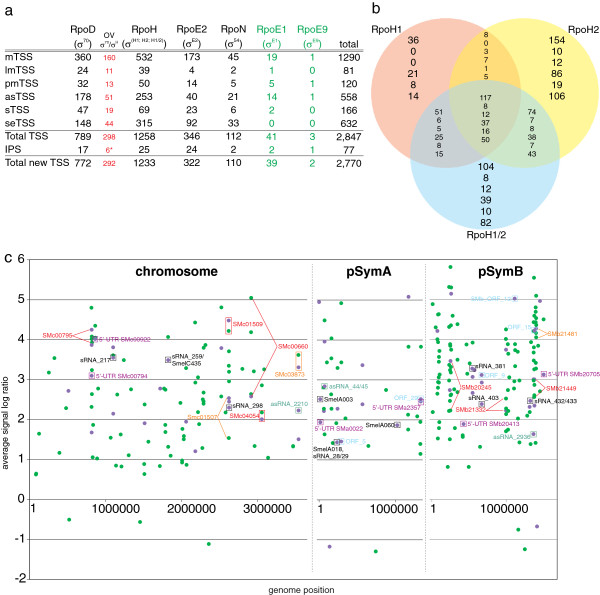
**Promoter predictions in *S. meliloti *and *rpoE2*-dependent genes/intergenic regions (IGRs).** (**a**) Number of predicted σ^70^, σ^H^, σ^E2^, and σ^54^ promoter sequences assigned to each TSS category. The number of promoter sequences used in the initial promoter search (IPS) is shown above the total number of newly identified promoters, and includes model, but not virtual promoters (Methods; Additional file 5: Table S23). Numbers in red represent promoter sequences that were assigned independently to at least two σ factors. The asterisk denotes 6 promoters that were used in the IPS for RpoH, but were also predicted for RpoD. (**b**) Venn diagram showing overlap among σ^H1^, σ^H2^, and σ^H1/H2^ promoter predictions. Each column of numbers represents the number of promoters assigned to each of the six transcript categories and are listed in the same order as in Figure 1a. (**c**) Plot of genes (green circles) and IGRs (purple circles) by replicon and position, showing decreased or increased expression with *rpoE2* overexpression. Black, *trans*-encoded sRNA; light green, *cis*-encoded antisense sRNA; red, gene and corresponding 5^′^-UTR; orange, gene and corresponding putative 5^′^-UTR; light blue, new ORF; purple, putative 5^′^-UTR only.

The ~17,000 TSS identified with RNAseq are an excellent resource with which to increase our knowledge about promoter properties in *S. meliloti*. As described above, previous promoter mapping has focused on regions upstream of a limited number of protein-coding genes. In addition, bioinformatics-based predictions have disregarded promoter motifs not associated with, or in antisense orientation to, protein-coding genes [27,62,65]. However in this study, TSS corresponding to ~3,400 asRNAs or sRNAs allowed us to directly identify 724 promoter sequences upstream of these genes, in addition to 1,371 promoters identified upstream of protein-coding (mTSS and lmTSS) genes (Figures 1c and 3a).

 The σ subunit of RNAP confers promoter specificity through interaction with conserved sequences upstream of TSS [3]. The *S. meliloti* genome encodes 15 σ factors: 14 of these belong to the σ^70^ family, and thus are predicted to recognize -35/-10-type promoters, while one (RpoN) belongs to the σ^54^ family and recognizes -24/-12-type promoters. Putative promoter consensuses have been identified corresponding to seven of the 15 *S. meliloti* σ factors (σ^E1^, σ^E2^, σ^E9^, σ^H1^, σ^H2^, σ^54^, and σ^70^), and for promoters recognized by both σ^H1^ and σ^H2^ (σ^H1/2^).

 Our promoter prediction procedure is illustrated in Additional file 1: Figure S3 and described in detail in Methods. Briefly, previously identified promoter sequences for σ^70^, σ^H1^, σ^H2^, σ^H1/2^, σ^54^, and σ^E2^ were used to calculate position-specific scoring matrices (PSSM) [27,62,65], which were then used in a genome-wide search for promoter motifs. The resulting motifs were correlated with the locations of all mapped TSS and deemed to be putative promoters if located in the appropriate position relative to the TSS (-24/-12 for σ^54^, -35/-10 for all others; Methods). For prediction of σ^E1^ and σ^E9^ promoters, we used a different approach. These two σ factors belong to the ECF class of σ factors, which are often autoregulatory on their own operons [66]. We had previously shown that expression of *rpoE1* and *rpoE4* is dramatically increased in *S. meliloti* 1021, compared to *S. meliloti* 2011 [67] due to a loss-of-function mutation in *ecfR1*, encoding the putative RpoE1 anti-σ factor (Krol and Becker, unpublished results) [68]. This suggested that RpoE1 autoregulates expression of its own operon and that of *rpoE4*. Examination of regions upstream of the TSS for these two operons revealed identical motifs (GAAT-N18-GTCT). This motif was used in global string searches to identify additional putative σ^E1^ motifs upstream of TSS (Methods). For RpoE9, we also performed string searching, but with the promoter motif recently predicted in [69], TGTCACA-N16-CGTC, which is located 9 nt upstream of the TSS we identified for the SMb20029-*rpoE9* operon.

Overall, we predicted 2,847 promoter sequences upstream of TSS (Figure 3a; Additional file 3: Tables S6-S13). σ^70^-dependent promoters make up the largest group of motifs (1,087, with 789 of these specific for σ^70^ only; Figure 3a; Additional file 3: Table S6). Similarity between the *S. meliloti* σ^70^ and σ^H^ promoter consensuses was previously noted [62], thus as expected, we found significant overlap (n=298) between the σ^70^ and σ^H^ promoter sets (Figures 3a and 3b). *E. coli* σ^70^ and σ^32^ promoter consensuses also share similar features [70], demonstrating that such overlap is not limited to *S. meliloti*, and that any *in silico* promoter prediction must be validated experimentally. In 2006, the first genome-wide prediction of *S. meliloti* σ^70^ promoter motifs provided a set of 117 experimentally validated (n=25) or computationally predicted (n=92) promoter motifs [65]*.* We confirmed that 55 of their 117 promoter sequences matched our TSS and σ^70^ promoter predictions. Ten promoter sequences identified in their study were not associated with mTSS in our study, but instead to TSS of other categories.

RpoH-dependent promoter motifs (σ^H1^, σ^H2^, σ^H1/2^) [62] were assigned to 1,556 unique TSS, of which 298 were also identified in searches with the σ^70^ promoter motif (Figure 3a; Additional file 3: Tables S7-S9). We identified extensive overlap among σ^H1^, σ^H2^, and σ^H1/2^ promoter predictions (Figure 3b; Additional file 3: Tables S7-S9). Barnett et al. [62] characterized the RpoH1 and RpoH2 regulons during heat shock and stationary phase growth: in addition to the experimental validation of 69 TSS downstream of putative RpoH-dependent promoters (51 of which were confirmed in this study), they predicted 75 putative promoters upstream of protein-coding genes [62]. Our data set validated ~59% (n=44) of these predicted promoters and added 647 previously unknown RpoH promoter motifs upstream of mRNA start sites (mTSS or lmTSS). Thirty-three σ^H^ promoter motifs are located upstream of *rpoH*-dependent genes, but were not previously identified by Barnett et al. [62]. Due to the similarity of RpoH and RpoD promoter motifs, the remaining set of 614 predicted RpoH promoters likely includes many false positives. RpoH-dependent regulation of the small non-coding RNAs SmelC781 and SmelC456 was proposed [62], and indeed, our analysis identified σ^H1^, σ^H2^, and σ^H1/2^ binding motifs upstream of SmelC781 and SmelC456 (Additional file 2: Table S2).

RpoN (σ^54^)-type σ factors are unique in that they require an activator to initiate transcription [3]. These activators, known as enhancer-binding proteins (EBP), interact with σ^54^ and bind DNA upstream of the RpoN-target promoters [3]. The *S. meliloti* genome is predicted to encode seven EBP (NifA, DctD, NtrC, NtrX, TacA, SMb20102, and SMb21200), but target genes have been identified for only the first three of these (reviewed in [25]). We identified RpoN-dependent promoter motifs (σ^54^) upstream of 112 TSS (Additional file 3: Table S10). Due to the lack of symbiotic samples in our RNA pools, we did not identify TSS upstream of the RpoN-dependent genes *nifA*, *nifB*, *nifH*, *glnII* and SMa0872. Only four of our predicted promoters (*dctA*, *glnB*, *glnK*, SMb20436) were previously characterized as RpoN-dependent or predicted by *in silico* methods [25]; therefore, our data provide a useful resource for the identification of additional RpoN-regulated genes and characterization of EBP.

For σ^E2^, 346 promoters were predicted (Additional file 3: Table S11), of which 47 were previously found by Sauviac et al. [27]. An additional 58 of these promoters were experimentally validated for *rpoE2*-dependence by microarray (Figure 3c; see below). Furthermore, we identified 41 putative σ^E1^ promoter motifs (Additional file 3: Table S12). In contrast, only two additional promoter motifs were predicted for σ^E9^ (Additional file 3: Table S13). This was not unexpected, as the RpoE9-type σ factors of *Rhodobacter sphaeroides* and *Bacillus licheniformis* were proposed to activate only their own operons [69].

Using our large set of newly predicted promoter motifs, we recompiled each of the consensus sequences for σ^70^, σ^54^, σ^E2^, σ^H1^, σ^H2^, and σ^H1/2^. As expected, the resulting motifs (Additional file 1: Figure S4) were similar to the previously reported consensus sequences [27,62,65]. As described before, the σ^70^ −10 region is poorly conserved in *S. meliloti*[62,65] (Additional file 1: Figure S4).

Global identification of promoter motifs in *S. meliloti* was hitherto limited due to limited knowledge about TSS of individual genes. Combining the TSS identified in this study with the previously identified sRNAs [17] provided a rich resource for identification of promoters and their assignment to σ factors. In summary, we were able to identify promoter motifs preceding ~17.5% (1,100 of 6,296; Additional file 3: Tables S6-S13) of known protein-coding genes [33] and 633 non-coding RNA genes (131 sRNA and 502 asRNA). We also identified 632 promoter motifs preceding 502 DNA regions corresponding to sense transcripts (distinct seTSS with one or more promoters). This finding supports the hypothesis that at least a small percentage of sense transcripts may represent intact transcripts, such as regulatory sRNAs or mRNAs that are transcribed from promoters internal to the predicted ORF [5].

### Transcription factor binding sites

While the σ subunit of RNAP is the primary means of conferring specific transcript initiation, it has long been known that various transcription factors interact with bacterial promoters to activate or repress transcription [3]. Activator-dependent promoters are common among those recognized by σ^70^ and often possess defective promoter elements [3]. Promoter searches using PSSM may fail to detect promoters possessing atypical core elements; therefore, understanding the mechanisms by which activators and repressors act at promoters is essential for a complete view of any transcriptional landscape. Unfortunately, such mechanisms, including specific transcription factor binding sites, are quite diverse and difficult to detect *de novo* on a global scale. However, TSS data can be used to provide information on promoter structure where binding sites are predicted or known, and to guide discovery of transcription factor binding sites where some functional information exists.

Additional file 4: Table S14 correlates previously reported transcription factor binding sites [34] with our TSS data. The group of predicted PhoB binding sites illustrates how TSS information can provide supporting empirical evidence for *in silico* binding site predictions. Under phosphate (P_i_) limitation, the PhoR histidine kinase phosphorylates its cognate response regulator, PhoB, which interacts with conserved motifs (PhoB boxes) upstream of genes involved in the P_i_ starvation response. While a large regulon of genes involved in *S. meliloti* response to P_i_ limitation has been identified, and several dozen PhoB boxes have been predicted [71,72], TSS have been determined for only three PhoB-dependent genes (*phoC*, *phoX*, and *pstS* in Additional file 2: Table S5). In *E. coli*, the PhoB box overlaps and replaces the −35 σ^70^ recognition elements of PhoB-dependent promoters [73]. Since the three S*. meliloti* PhoB-dependent genes with mapped TSS appeared to have poor −35 σ^70^ recognition elements overlapping with PhoB motifs, we matched the locations of previously predicted PhoB boxes to our TSS. Of the 33 genes with predicted PhoB boxes and TSS in this study, 16 overlapped the −35 motif (i.e. were located 22 to 25 nt upstream of the TSS); and therefore, are strong candidates for PhoB binding sites (Additional file 4: Table S14). Additional PhoB boxes are located farther upstream of TSS; these may be targets for other types of regulation, for example, PhoB-mediated repression, as was proposed for SMc02862 [74].

An example of how TSS data may guide binding site discovery is illustrated by the example of CtrA, a global cell cycle regulator. CtrA is autoregulatory upon its own promoter, which contains five verified CtrA binding sites (Figure 1d) [38]. In contrast to *C. crescentus*, where the CtrA regulatory circuit is well characterized [75], little is known about CtrA targets in *S. meliloti*. We used PSSM to locate additional putative CtrA binding sites upstream of *S. meliloti* protein-coding genes and compared these to our TSS data (Methods). We identified 175 putative binding motifs, representing 158 genes: ~70% of the motifs are located upstream of TSS or overlap TSS (Additional file 4: Table S15). We confirmed CtrA motifs upstream of TSS corresponding to *S. meliloti* cell cycle-related genes previously predicted to possess such motifs (*minC*, *chpT*, *rcdA*, *pleC*) [76,77], and identified CtrA motifs upstream of *rpoD* and *ftsE*, previously shown to bind CtrA in *Brucella abortus*[78]. Other *S. meliloti* genes with CtrA motifs that are plausibly cell cycle regulated or were shown to be direct CtrA targets in *C. crescentus* include *podJ*, *mraZ*, *metK*, *clpP2*, *ftsK*, *flaA*, *flaC*, *flaD*, *mcpE*, *mcpY*, and SMc00651 (Additional file 4: Table S15). Our data also suggest potential regulatory mechanisms; for example, *S. meliloti gcrA*, an ortholog of *C. crescentus gcrA* encoding a master cell cycle regulator that activates *ctrA* transcription [75], is transcribed divergently from *argD* and possesses a strong CtrA motif overlapping the *gcrA* TSS, but on the opposite strand. A weaker CtrA motif that did not make our cutoff (TAA-N_7_-TTACT; P-value = 3.4×10^-4^) is located on the same strand as the *gcrA* TSS and almost completely overlaps the strong motif. The regulatory significance of this arrangement is unknown, but we speculate that CtrA may mediate a complex mechanism of both activation and repression at the *gcrA* promoter.

### *de novo* promoter motif discovery

Of the 4,925 protein-coding genes for which we did not identify a promoter, 3,468 lacked TSS with ≥30 reads. The remaining 1,457 genes had one or more TSS (mTSS or lmTSS), but lacked an upstream promoter prediction. These may represent genes transcribed by σ^70^, σ^54^, σ^E1^, σ^E2^, σ^E9^, σ^H1^, or σ^H2^, but whose promoters contain atypical core motifs, or genes whose promoters are recognized by one more of the other eight *S. meliloti* σ factors (σ^E3^, σ^E4^, σ^E5^, σ^E6^, σ^E7^, σ^E8^, σ^E10^, and σ^fecI^). In order to determine whether any of these genes lacking a promoter prediction share similar, as yet to be identified promoter motifs, we extracted sequences 40 nt upstream of high coverage TSS (≥300 reads) and used them as input for MEME [79]; Methods). Using this set of 482 upstream sequences, we identified three motifs with E-values of < 1 (Additional file 1: Figure S5). Motif 1 was identified upstream of 159 genes and consists of a conserved upstream TTG and a conserved downstream A residue (Additional file 1: Figure S5). In addition to genes lacking an annotated function (~40%), the list includes genes involved in housekeeping, transport, metabolic, and regulatory functions (Additional file 4: Table S16). Therefore, we speculate that Motif 1 corresponds to genes transcribed by RpoD that possess a conserved TTG motif, but lack other features of the RpoD consensus sequence (Additional file 1: Figure S4). Motif 2 contains a central TTTGTTAACCAT sequence and was identified upstream of 19 genes (Additional file 1: Figure S5; Additional file 4: Table S16), 84% of which were previously shown to have high expression in rich medium, yet decreased expression in nodule bacteria and during heat shock [62,80]. Motif 2 does not appear to match any previously identified *S. meliloti* promoter motifs or any motifs in the manually curated prokaryotic PSSM database, RegTransBase [81]. Motif 3 (TTCA-N7-TTCA) was identified upstream of 12 genes (Additional file 1: Figure S5; Additional file 4: Table S16), five of which were previously identified as FeuP-dependent [82]. FeuP encodes a response regulator required for bacterial invasion of root nodules and activation of at least 14 genes [82], including *ndvA* encoding a cyclic σ-glucan exporter, and *feuN*, a negative modulator of the FeuPQ pathway that is predicted to be cotranscribed with *feuPQ* (Additional file 4: Table S17) [83]. We checked whether Motif 3 was present upstream of the remaining nine FeuP-dependent genes: three (including *feuN*) had motifs in appropriate distance upstream of TSS and three had motifs upstream of ORFs for which TSS were not identified (Additional file 4: Table S17). Therefore, we suggest that Motif 3 is a candidate FeuP binding site. In sum, our identification of three novel motifs upstream of TSS demonstrates the utility of global TSS data as a discovery tool.

### The *S. meliloti* RpoE2 (σ^E2^) regulon

 The *S. meliloti* ECF σ factor, RpoE2 (σ^E2^), responds to heat shock, osmotic stress, oxidative stress, and carbon/nitrogen starvation during stationary phase growth [27,84], activating expression of at least 45 genes [27]. Most α-proteobacteria possess RpoE2-like σ factors and their role was proposed to be analogous to that of RpoS in *E. coli*[85]. The activity of σ^E2^ is controlled by a partner switching mechanism, which includes negative regulation via two paralogous anti-σ factors, RsiA1 (SMc01505) and RsiA2 (SMc04884), and positive regulation via two anti-anti-σ factors, RsiB1 (SMc01504) and RsiB2 (SMc00794) [27,86]. RsiB1 and RsiB2 each contain a C-terminal receiver domain, which is phosphorylated by an unidentified histidine kinase(s), allowing the N-terminal ECF σ-like domain to interact with RsiA1 or RsiA2, thereby relieving inhibition of σ^E2^[86].

A previous study identified *rpoE2*-dependent genes in *S. meliloti* 1021 by comparing transcription profiles of an *rpoE2* mutant and wild type during heat shock [27]. To identify additional RpoE2 target genes, independent of any specific stress condition, and to verify RpoE2-dependent regulation of genes associated with putative σ^E2^ TSS identified in this study, we used custom Affymetrix GeneChips to obtain transcription profiles of *S. meliloti* strains either overexpressing *rpoE2* from an inducible promoter or carrying the empty vector (Methods). As mentioned above, * S. meliloti* 1021 has a defective *ecfR1* gene, resulting in high constitutive RpoE1 (σ^E1^) activity. Sigma factors compete with each other for RNAP [3]; therefore, to maximize the amount of RNAP-σ^E2^, and presumably enhance identification of σ^E2^ targets, we performed our experiments in a strain containing a functional *ecfR1* gene (CL150; Methods).

 Our analyses revealed that 202 protein-coding genes displayed more than twofold increased transcript abundance in the strain overexpressing *rpoE2*, whereas three mRNAs showed lower abundance (Additional file 5: Table S18). The majority of the protein-coding genes (n=150) lack a predicted function (Additional file 5: Table S18). About 95% of *rpoE2*-dependent transcripts previously identified by microarray [27], showed increased expression in the *rpoE2* overexpressing strain. In addition, we identified 161 new *rpoE2*-dependent protein-coding genes (fold change ≥2), 41 of which confirm previous *in silico* predictions [27]. Overall, ~72% of *rpoE2*-dependent protein-coding genes had one or more mapped TSS in our RNAseq data (Additional file 5: Tables S18 and S20).

**Figure 4:**
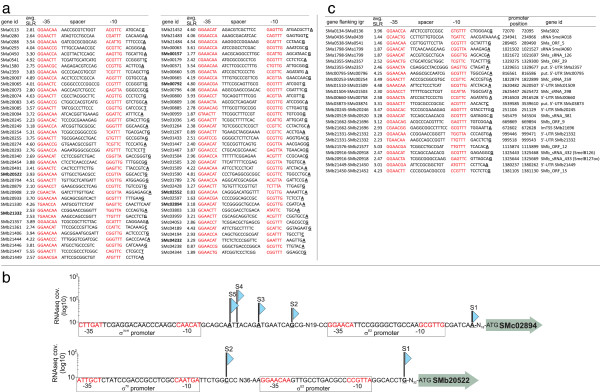
**Promoter predictions for *rpoE2*-dependent genes and intergenic regions (IGRs).** (**a**) Protein-coding genes whose transcripts showed increased abundance when *rpoE2* was overexpressed, and for which an mTSS and σ^E2^ promoter motif was assigned. Genes in bold were predicted to possess σ^70^ or σ^H^ promoters, in addition to the σ^E2^ promoter. (**b**) Upstream regions of SMc02894 and SMb20522 showing transcription start sites (indicated by blue triangles) and putative promoters. The height of each TSS symbol corresponds to the log_10_ coverage for mapped reads (y-axis). **(c)** IGRs whose transcripts showed increased abundance when *rpoE2* was overexpressed.

 As expected, expression of regulatory genes *rpoH2* (SMc03873), *rpoE5* (SMb21484), *rsiA1* (SMc01505), and *rsiB1* (SMc01504) was *rpoE2*-dependent; moreover, each of these genes has an mTSS downstream from an RpoE2-dependent promoter (Figure 4a; Additional file 5: Table S19). In addition, we identified TSS with σ^E2^ motifs upstream of two operons encoding *rpoE2*-dependent response regulator-histidine kinase pairs: SMa0113/114 and *exsFG* (SMb20933/934; Additional file 5: Table S19). These observations demonstrate the complexity of the RpoE2 regulatory circuit and suggest that *rpoE2* overexpression may result in multiple, secondary regulatory effects. Thus, 76 *rpoE2*-dependent protein-coding genes possess a TSS preceded by a σ^E2^-binding motif: these are likely directly controlled by σ^E2^, while transcription of the remaining genes likely depends on other σ factors (Figures 4a and 4c, Additional file 5: Table S19). For example, 22 *rpoE2*-dependent operons were previously shown to be *rpoH*-dependent [62]. Fifteen (68%) of these had an RNAseq TSS preceded by a promoter prediction for RpoE2 (n=5), RpoH (n=9), or both (n=1) (Additional file 5: Tables S18 and S19).

 In addition to detecting mRNAs corresponding to protein-coding genes, our custom Affymetrix chip detects transcriptional activity for IGRs ≥ 150 nt. The strain overexpressing *rpoE2* showed increased hybridization, compared to the control strain, to 63 probe sets, corresponding to 56 IGRs, and decreased hybridization to two probe sets (Figure 3c; Additional file 5: Tables S18 and S20). Our RNAseq approach detected transcripts corresponding to 43 of these IGRs. About 60% of IGRs showing *rpoE2*-dependent hybridization are adjacent to *rpoE2*-dependent protein-coding genes, and therefore likely represent 5^′^- or 3^′^-UTR of those genes (Figure 3c; Additional file 5:Tables S18 and S20). IGRs corresponding to 5^′^-UTRs of six genes SMa0022, SMa2357, SMb20413, SMb20705, SMc00794, and SMc00922 showed increased expression in the *rpoE2* overexpressing strain that did not correlate with that of their corresponding coding regions (Figure 3c; Additional file 5: Table S18); however, we confirmed *rpoE2* dependence for three of these by 5^′^ RACE mapping (SMa2357, SMb20705, SMc00922; see below). Five protein-coding genes, newly identified in this study, correlated with *rpoE2*-dependent IGRs identified by Affymetrix analysis (Figure 4c; Additional file 5: Table S20).

 σ^E2^ promoter motifs were predicted upstream of 23 sRNAs (Figure 3a, Additional file 5: Table S20). Our microarray data provides evidence for *rpoE2*-dependent transcription in eight IGRs where sRNA genes are located: SmelA018, SmelA060, SMa_sRNA_126, SMb_sRNA_381, SMb_sRNA_432 (SmelB126), SMb_sRNA_433 (Smel-B127ov), SMc_sRNA_259, and SMc_sRNA_298 (Figures 3c and 4c; Additional file 5: Table S20). This suggests that sRNA mediated post-transcriptional control is part of the RpoE2 regulatory circuit.

**Figure 5:**
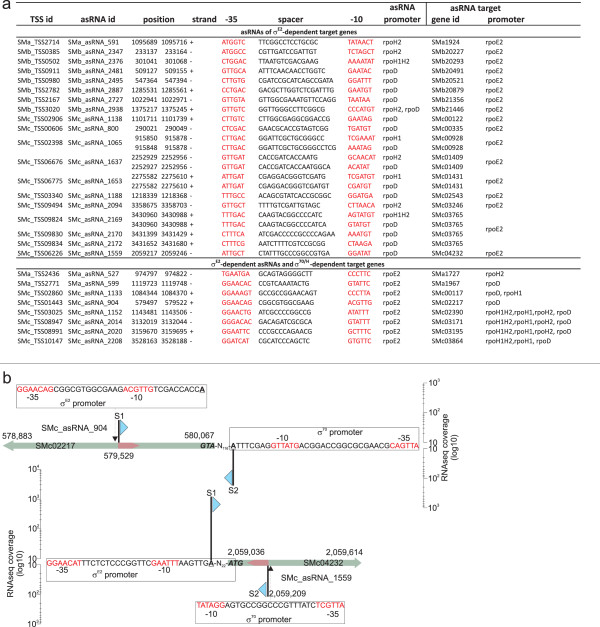
**Identification of potential interactions between antisense RNAs (asRNAs) and transcripts of different σ factor regulons.** (**a**) σ^70^-, σ^H^-, and σ^70/H^-dependent asRNAs located in antisense to σ^E2^-dependent target genes (top panel) and σ^E2^-dependent asRNAs located in antisense to σ^70^-, σ^H^-, and σ^70/H^-dependent target genes (bottom panel). (**b**) Genomic location of the asRNAs SMc_asRNA_904 and SMc_asRNA_1559 showing transcription start sites (indicated by blue triangles) and putative promoters. The height of each TSS symbol corresponds to the log_10_ coverage for mapped reads (y-axis).

σ^E2^ promoter motifs were identified upstream of 40 asRNAs (Figure 3a; Additional file 3: Table S11). At the post-transcriptional level, *cis*-encoded asRNAs and *trans*-encoded sRNAs may play a role in complex regulatory circuits by allowing for interference between transcripts of different σ factor regulons. For example, eight *rpoE2*-dependent asRNAs are located in antisense to genes are predicted to be controlled by σ^70^, σ^H^ or both (Figure 5a). Figure 5b shows an example for the σ^E2^-dependent SMc_asRNA_904, which may interact with the σ^70^-dependent SMc02217 mRNA. In addition, 70 asRNAs were found in antisense to *rpoE2*-dependent genes. We predicted binding motifs for σ^70^, σ^H^, or both, upstream of 17 of these asRNAs. An example is the σ^70^-dependent SMc_asRNA_1559, which may interact with the *rpoE2*-dependent SMc04232 mRNA (Figures 5a and 5b). These observations strongly support the hypothesis that asRNAs belonging to a distinct σ factor regulon can interact with targets of different σ factor regulons.

### 5^′^ RACE mapping of *rpoE2*-dependent transcripts

To confirm RNAseq and Affymetrix data, we chose 26 genes for 5^′^ RACE (random amplification of cDNA ends) mapping (Methods). For these experiments, we used the same RNA samples as were used for the Affymetrix analyses. Because IGR probe sets on our custom Affymetrix GeneChip are a low-resolution means of delineating transcript structure, we examined TSS and *rpoE2* dependence for putative sRNAs, newly identified ORFs, and 5^′^-UTR detected by these IGR probe sets. Also, since RpoE2 was shown to activate expression of *rpoH2*[27], we tested seven *rpoE2*-dependent genes whose expression was previously shown to be *rpoH*-dependent [62]. As controls, we included two regulatory genes whose expression was not *rpoE2*-dependent, *rpoH1* and *rsiA2*. Results of 5^′^ RACE mapping are provided in Additional file 5: Table S21, and gel photos showing results for four representative transcripts are shown in Additional file 1: Figure S6. Of the 26 RACE mapped genes, 24 had TSS in the RNAseq data, and all but three of these TSS matched our RACE determined start sites exactly. *rpoE2* dependence was confirmed qualitatively by 5^′^ RACE for all genes tested except for SMb20413, and as expected, *rpoH1* and *rsiA2*. Most mapped promoters had upstream matches to the RpoE2 promoter consensus sequence, thus the corresponding genes are likely direct targets of RpoE2. Two genes previously identified as *rpoH*-dependent (SMc01507 and SMc03968; [62]), had RpoH-like promoters.

## Conclusions

To our knowledge, this study reports the first genome-wide RNAseq identification of TSS in an α-proteobacterium. Our approach defined a set of 17,001 TSS that provides mRNA start sites for ~45% of *S. meliloti* protein-coding genes and identifies new ORFs and ncRNAs, including asRNAs complementary to about one third of protein-coding genes. Over 2,000 TSS are preceded by at least one predicted promoter sequence, representing potential target promoters for nearly half of the known *S. meliloti* σ factors. As promoter consensus sequences become available for the remaining σ factors, our data set can be used to identify target genes of these σ factors. Also, we demonstrated how TSS identification on a global scale aids in discovery of novel promoter motifs and transcription factor binding sites. Combined with our Affymetrix GeneChip analyses, the RNAseq data expand the known regulon of the σ factor, RpoE2, and identify candidate ncRNAs that may be involved in the *S. meliloti* general stress response. In sum, our work provides a rich resource for continued study of *S. meliloti* gene expression and regulatory circuits, as well as a foundation for comparative studies of transcription in other α-proteobacterial species.

## Methods

### Strains and growth conditions for RNAseq experiments

 The sequenced reference strain, *S. meliloti* strain 1021 [20,87], and *S. meliloti* strain 1021*expR*^+^ were used for the RNAseq experiments. *S. meliloti* strain 1021*expR*^+^ was constructed by replacing *expR*, disrupted in 1021 by an insertion element [88], with a functional *expR* gene. To repair *expR*, the corresponding genomic sequence of strain Rm8530 [89] was amplified using primers 5^′^-ACACAAGCTTCTTCTGAACGGCGTATTCACA and 5^′^-TGATGAATTCCATTCCGTCGGCGAGATAGT and cloned into the *Hind*III and *Eco*RI restriction sites of pK18mobsac [90]. The construct was introduced into the genome of Rm1021 by conjugation. Transconjugants were subjected to sucrose selection on LB (Luria-Bertani) agar plates [91] with 10% sucrose, which allowed for selection of the 1021*expR*^+^ strain.

 For large-scale identification of TSS in the genome of *S. meliloti* 1021, RNA samples representing 16 different growth and stress conditions, each in three biological replicates, were purified and pooled. Unless stated otherwise, 50 ml cultures of *S. meliloti* strains 1021 and 1021*expR*^+^ were inoculated to a starting OD_600_=0.02 in 250 ml flasks and incubated at 30°C with shaking (180 rpm) in TY [92], VMM [93] or MOPS-MM medium [94], supplemented with 8 μg/ml nalidixic acid.

Exponential and stationary phase RNA samples were purified from *S. meliloti* 1021 cultures grown in TY (OD_600_=0.8 or 3), VMM (OD_600_=0.6 or 1.3), and MOPS-MM (OD_600_=0.5). Exponential phase cultures of *S. meliloti* 1021*expR*^+^ were grown in MOPS-MM (OD_600_=0.5). For exposure to various environmental stresses, the following modifications were made. All stress treatments were applied to strain 1021 only, unless noted otherwise:

Temperature shifts for heat (40°C) and cold shock (20°C) were applied for 30 min to cultures grown in TY.

 Exposure to microoxia was performed by flushing TY-grown cultures with pure nitrogen (N_2_) for 1-minute, followed by incubation of cultures under a N_2_ atmosphere for 45 minutes.

 Salt stress was carried out by adding sodium chloride (NaCl) to exponential phase, VMM-grown cultures at final concentration of 0.4 M for 20 minutes. Oxidative stress exposure was performed similarly, except instead of NaCl, H_2_O_2_ was added to a final concentration of 10 mM.

Exposure to acid and alkaline stress was as follows: cultures were grown to an OD_600_ of 0.6, centrifuged, and the resulting cell pellets resuspended in VMM modified by addition of either HCl to pH 5.8 or NaOH to pH 8.5.

Cultures were starved for nitrogen or carbon by growing them to an OD_600_ of 0.6 in replete MOPS-MM, followed by centrifugation and resuspension of the cell pellets in MOPS-MM lacking either a nitrogen or carbon source [95]. *S. meliloti* 1021 and 1021*expR*^+^ cultures were subjected to phosphate limitation in MOPS-MM with 0.1 mM phosphate as described previously [71].

 For induction with the flavonoid luteolin, cultures were grown in TY to an OD_600_ of 0.8, luteolin was added to a final concentration of 10 μM, and cells were harvested after 4 hours incubation.

### RNA preparation for RNAseq

Total RNA was isolated using miRNeasy Mini kits and a QIAcube automated nucleic acid preparation platform, according to manufacturer’s instructions (Qiagen). RNA samples were further purified by phenol/chloroform/isoamyl alcohol extraction and ethanol precipitation. The integrity of all RNA samples was confirmed using a RNA 6000 Nano Kit with a Bioanalyzer 2100 (Agilent Technologies).

### cDNA library preparation and RNAseq

 The pooled RNA sample was sequentially treated with two enzymes: (i) terminator 5^′^-phosphate-dependent exonuclease (TPE; Epicentre), which specifically degrades RNA species with 5^′^ monophosphates (processed transcripts), and (ii) tobacco acid pyrophosphatase (TAP; Epicentre), which prepares the TPE-resistant transcripts from (i) for adapter ligation by removing pyrophosphates from their 5^′^ ends. Subsequently, an adapter comprised of sequences complementary to the sequencing and amplification primers was ligated to the 5^′^ phosphate of the pretreated mRNA. First-strand cDNA synthesis was then performed with an N6 randomized adapter primer and M-MLV-RNase H reverse transcriptase (New England Biolabs). The resulting cDNA was PCR-amplified in 24 cycles. The primers used for PCR amplification were designed for amplicon sequencing according to Illumina/Solexa guidelines. The following adapter sequences flanked the cDNA inserts: 5^′^ end, 5^′^- AATGATACGGCGACCACCGACAGGTTCAGAGTTCTACAGTCCGACGATCTCCA-3^′^ and 3^′^ end, 5^′^- CAAGCAGAAGACGGCATACGATCAGGCAGAGGACGAGAA-3^′^ (Illumina adapter sequences are underlined). Prior to sequencing, double stranded cDNA was enriched for fragments of 300 to 500 nt (the optimal range for our sequencing method) by size fractionation. RNAseq was performed on the Illumina HiSeq 2000 sequencing system by GATC Biotech (Konstanz, Germany).

 We note that while our procedure resulted in an enrichment of primary 5^′^ ends, such enrichment is never complete. Hence, a detectable proportion of a given abundant transcript with a processed 5^′^ end can survive the enrichment procedure. In addition, the specific tertiary structure of some processed RNAs may result in their 5^′^ ends being stably protected against TPE degradation. Therefore, for some genes, both primary and processed transcripts may be represented in our data set.

### Computational methods

#### Identification of transcription start sites (TSS)

 Sequences of the *S. meliloti* 1021 genome, accession numbers NC_003037 (pSymA), NC_003047 (chromosome), NC_003078 (pSymB), and gene annotations, including the set of sRNAs previously identified by 454 sequencing [17], were downloaded from NCBI and the RhizoGate Portal [33], respectively.

 Sequencing reads from the cDNA library were processed and mapped to the *S. meliloti* 1021 genome using segemehl [96]. From an initial set of 37,792,608 reads, 35,043,949 reads ≥ 18 nt were mapped to the reference sequence. To enrich for the number of reads originating from a TSS (the 5^′^ end of a transcript), only reads that mapped to a unique position in the genome, and without mismatches in the first three bases of the 5^′^ end, were retained for downstream analysis. This resulted in a final set of 11,230,166 reads. The overall workflow is outlined in Additional file 1: Figure S1.

 The next step in the workflow was to identify TSS. We assembled reads into TSS contigs, assemblies of at least 30 mapped reads sharing a common 5^′^ end. Thus, the 5^′^ end of a TSS contig constitutes a TSS, while the 3^′^ end of the longest read determines the end position of the TSS contig. If the distance of two TSS positions differed by less than 3 nt, they were treated as a single TSS and merged. Altogether, we identified 17,001 TSS contigs: 15,056 that originated from a single TSS contig, and 1,945 that were assembled from two or more TSS contigs. TSS were named using the scheme: SMx_TSS_nnnn where x = A, B or C and denotes a location on pSymA, pSymB or the chromosome, respectively.

 Based on a minimal transcriptional unit (MTU) model, we derived six classes of TSS: lmTSS, mTSS, pmTSS, seTSS, asTSS, and sTSS (see below). These classes reflect the relative position and orientation of a TSS contig to its genomic context. The TSS classes are organized hierarchically to guarantee that TSS contigs that meet definitions of multiple classes are assigned to a single class.

The MTU of a gene is divided in three consecutive regions: 5^′^-untranslated region (UTR), coding sequence (CDS), and 3^′^-UTR. Nucleotide numbering starts with 1 at the first nucleotide of the 5^′^-UTR. The size of the coding segment is delimited by the position of its start and stop codon. In contrast, we used fixed length thresholds for the UTRs, which represent the minimal required sizes of ribosome binding sites (RBS; 14 nt) and terminator hairpins (20 nt) within the 5^′^-UTR and the 3^′^-UTR, respectively. The promoter region upstream of the MTU comprises nucleotides from position -40 to -1.

### TSS class definitions

#### mTSS

 These TSS are probably associated with mRNAs. To associate identified TSS with protein-coding genes, the defined minimal length of 14 nt of the 5^′^-UTR, the defined minimal length of 40 additional nt for the promoter region, and the length range of the sequence contigs assembled from our data were taken into account. Contigs ranged from 87 to 108 nt. If a contig was in the same orientation and overlapped the minimal region of 14 plus 40 nt upstream of the start codon, we assumed that it is more likely associated to an mRNA than to a *trans*-encoded sRNA and therefore classified its prominent 5^′^ end as an mTSS.

Also, to identify additional mTSS, we included *cis*-encoded mRNA leader sequences from the 454 RNAseq data [17] in our analyses (see below). Thus, a TSS contig, which is located upstream of a promoter region and without overlapping it, is categorized as mTSS, if there exists a *cis*-encoded mRNA leader sequence for this MTU that starts at the same position or upstream of the mTSS.

#### lmTSS

These TSS are associated with leaderless transcripts and match the first nucleotide of the corresponding AUG start codon.

#### pmTSS

These represent a class of putative mTSS where it is uncertain if the TSS belongs to an mRNA or to the transcript of an sRNA. This classification is used because TSS located over 300 nt away from the corresponding start codon are rarely observed. TSS contigs of pmTSS do not overlap with any MTU in sense and antisense and are located at most 300 nt upstream in same orientation of a start codon.

#### seTSS

 Sense TSS must meet two criteria: the TSS is located within an MTU, and has the same orientation as the associated MTU. This is true for all TSS within an MTU except for those that meet the definition of the lmTSS class.

#### asTSS

These TSS represent *cis*-encoded antisense RNAs. In contrast to seTSS, the asTSS contigs overlap MTUs in antisense orientation.

#### sTSS

These TSS represent *trans*-encoded sRNAs located in IGRs. Therefore sTSS contigs do not overlap any MTU. In the case where an MTU is located downstream, in the same orientation, a TSS is classified as an sTSS only if the distance between start codon and TSS exceeds the pmTSS threshold of 300 nt.

### Definition of *trans*-encoded sRNAs and *cis*-encoded antisense RNAs

 After categorizing the TSS, we merged overlapping TSS contigs of sTSS and asTSS. The resulting *trans*-encoded sRNA and *cis*-encoded antisense RNA transcripts are named according to the following conventions, SMx_sRNA_n and SMx_asRNA_n, respectively.

### Identification of new sRNAs from 454 RNAseq data

Schlüter et al. [17] used a 454 RNAseq approach to identify non-coding RNAs shorter than 350 nt. In that study, 454 contigs were categorized as follows: *trans*-encoded sRNA, *cis*-encoded mRNA leader, sense sRNA, and *cis*-encoded antisense RNA. For this study, we reanalyzed the 454 RNAseq data using less stringent parameters to identify additional sRNAs. We defined a 454 contig as a region with continuous coverage of at least 5 reads and a length between 50 and 350 nt. From this initial set, we chose only those 454 contigs whose 5^′^ end matched a TSS from this study. Schlüter et al. [17] adopted a naming convention of SmelXnnn for their sRNAs; in order to distinguish our newly identified sRNAs we inserted an R (SmelXRnnn), indicating that relaxed parameters were applied.

### Comparative gene prediction

If the TSS of an organism are known, one may guide the gene annotation process by using the genomic coordinates of the TSS as constraints for gene prediction. Here, we combined our TSS data with RBS (ribosome-binding site) and open reading frame (ORF) predictions, and carried out protein similarity searches to improve the *S. meliloti* genome annotation by finding new genes and correcting positions of start- and stop-codons. Our procedure is presented in Additional file 1: Figure S2.

For each TSS transcript, T, we performed the following steps: First, the set of putative ORFs that overlap with T were identified. Then, we distinguished between two kinds of ORFs, depending on the translation initiation mechanism: leaderless ORFs and Shine-Dalgarno (SD) initiation ORFs (sdORFs). While a leaderless ORF possesses an AUG start codon and its 5^′^ end coincides with the TSS, sdORFs are preceded by a leader sequence, with the TSS as the 5^′^ end. Since the leader sequences contain an RBS, we set the minimum length threshold to 14 nt. In the next step, all ORF sequences were translated and matched against the NCBI non-redundant (nr) protein database using BLASTP. BLASTP hits with an E-value < 10^-5^ were selected if the BLASTP alignment covered the query sequence completely and if the matching protein and the query were of similar length. For sdORFs we used free_align to predict RBS on the leader sequences. free_align identifies an RBS as the energetically most favorable hybridization site of a target leader sequence with the 3^′^ tail of a 16S RNA by applying free energy calculations. Following Starmer et al. [97], we set the energy cutoff for a RBS to −3.4535 kcal/mol. Subsequently, we classified sdORFs with RBSs and leaderless ORFs as either new ORFs or reannotated ORFs. New ORFs did not overlap more than the start or stop codon of existing annotated ORFs. If an ORF *O* shared either the 5^′^ or the 3^′^ end, denoted by a start or stop codon, respectively, with a gene *G*, *O* was classified as a reannotated ORF. ORFs that did not fall into these two categories were discarded, as the purpose of the ORF prediction procedure was not to replace current gene annotations with another gene annotation having a different gene product. New ORFs were named following the scheme, SMx_ORF_n. All new ORFs and reannotation ORFs were included in the GenDB *S. meliloti* 1021 database [33].

### Identification of promoter motifs

#### Promoter consensus determination and *in silico* genome-wide predictions

Parameters such as spacer threshold and promoter element size used to search for putative promoter sequences specific for RpoD, RpoE2, RpoE1, RpoE9, RpoH1, RpoH2, RpoH1/2, and RpoN are listed in Additional file 5: Table S22. In contrast to previous studies, all regions upstream of TSS were included in our analyses, regardless of its position in an IGR or a CDS. To discover promoter sequences, we followed two different strategies, depending on available input data: string-based or profile searching (see below).

### String-based promoter search for RpoE1 and RpoE9 promoters

We used the consensus motifs, GAAT-N18-GTCT (RpoE1; unpublished observations; [68]) and TGTCACA-N16-CGTC (RpoE9; [69]), to perform pattern matching on the *S. meliloti* 1021 genome. We scanned for putative promoters that had no more than a single mismatch with respect to the consensus, a spacer length in the range specified in Additional file 5: Table S22, and were located upstream of a TSS with minimum and maximum distance of 5 nt and 12 nt from the end of the promoter, respectively.

#### Profile search of promoters

 Previously identified promoter sequences were used as input for the prediction of RpoD- (n=25), RpoH1- (n=20), RpoH2- (n=11), RpoH1/H2- (n=14), and RpoN- (n=8) specific promoters [62,65,98]. For the RpoN promoter search model, we excluded two that were previously reported [98] because one was shown to be RpoD-like and the other was not found in *S. meliloti* strain 1021. For RpoE2 promoters, only one RpoE2-dependent TSS, that of the *rsiA1*-*rpoE2* operon, had been mapped prior to this study [86]; therefore, we used a set of 35 putative promoters predicted previously by aligning upstream regions of a set of *rpoE2*-dependent genes [27]. For purposes of our study, input promoters were designated as either “virtual”, if they had been previously reported in the literature, but no TSS corresponding to the promoter was present in our RNAseq data, or as “model” if an RNAseq TSS was located an appropriate distance downstream (Additional file 3: Tables S6-S11 and Additional file 5: Table S23).

 In view of the sparse promoter data, we implemented an iterative two-phase procedure to both identify the global promoter architecture and to refine the promoter consensus. The main analytical steps of our promoter prediction workflow are outlined in Additional file 1: Figure S3.

 Before beginning Phase 1, we extended the set of input promoter sequences by searching for promoter regions identical to the −35 and −10 elements of each input promoter, and within an appropriate distance of a TSS (denoted “extended model ” in Additional file 3: Tables S6-S11).

In Phase 1, promoter candidates were identified by means of position-specific scoring matrices (PSSMs). A PSSM is the probabilistic representation of a promoter motif; it summarizes a conserved pattern by modeling nucleotide variability at each position of the motif. We conducted a profile search for seed promoter sequences, using the initial PSSMs obtained with the input promoter sequences. First, −35 and −10 elements were extracted from the input promoter sequences, aligned, and separate PSSMs constructed from the alignments with a pseudocount=1. After constructing PSSMs, we performed a profile search on the *S. meliloti* genome for putative promoter elements using PoSSuMsearch [99], Then, based on the promoter structure we computed chains of collinear −35 and −10 elements, such that a −35 element is located upstream of a −10 element and the distance between the elements complies with the spacer length threshold (Additional file 5: Table S22). Central to the promoter prediction process was the integration of TSS and 454 contigs [17] with promoter candidates. Promoter candidates were retained only if they were located 5 nt to 12 nt upstream of a TSS or 454 contig. The last filter step of Phase 1 discarded all promoters that had promoter elements with P-value > 0.005 or that were associated with seTSS (sense sRNAs) because such predictions were more likely to be false positives. This ensured that only promoters that were good representatives of their respective promoter motif were combined with the input promoter sequences to form a new set of seed promoter sequences (denoted “new from initial search” in Additional file 3: Tables S6-S11).

In Phase 2, we started with the new set of promoter sequences obtained from Phase 1. All steps of Phase 1 were then repeated with this new set and the same parameters, except for the last filter step. Since the aim of Phase 2 was to obtain a comprehensive set of promoters and to elucidate promoter architecture, we lowered the restrictions by allowing all classes of TSS to be associated with promoters and increased the P-value threshold to 0.02 (Additional file 1: Figure S3). The additional promoters identified in Phase 2 are denoted “second search” in Additional file 3: Tables S6-S11.

### Identification of CtrA binding motifs

To identify putative CtrA binding sites upstream of *S. meliloti* protein-coding genes, we used a matrix-based search method, RSA-tools-matrix-scan [100]. The PSSM used for searching included sequences of five experimentally verified CtrA binding sites upstream of *ctrA* (consensus=TAA-N_7_-TTAAC; [38]). Although one of these binding sites has 6 nt spacing, we searched only for motifs with 7 nt spacing because CtrA binding sites with spacing ≠ 7 nt showed low affinity binding in *C. crescentus*[101]. We searched the direct strand of DNA upstream of all ORFs (pseudo-frequencies=0.01) and retained those hits that did not overlap ORFs and that had a positive weight score and P-value ≤ 1×10^-4^.

### *de novo* promoter motif discovery

For *de novo* promoter motif discovery we compiled a set of 482 sequences representing 40 nt upstream of mTSS and lmTSS that were not assigned to a σ factor and had a coverage of at least 300 reads (10 fold of the coverage threshold used to identify TSS). The sequences were used as input for MEME (multiple em for motif elicitation) version 4.8.1 [79], with the following parameters: -dna -mod anr -minw 18 -maxw 40 -minsites 10. Motifs with an E-value of <1 were examined further.

### Affymetrix GeneChip analysis and 5^′^ RACE mapping

To identify genes whose expression was *rpoE2*-dependent, we used Affymetrix GeneChip analysis to compare two strains: CL150 pCAP11 and CL150 pF1084. CL150 is an *S. meliloti* 1021 strain in which two point mutations were corrected by gene replacement: *ecfR1*, encoding the RpoE1 anti-σ factor, and *pstC*, encoding a subunit of the high affinity phosphate transporter [37]. To repair *ecfRI*, the corresponding genomic sequence of strain Rm2011 [67] was amplified using primers 5^′^-CTCGAGCTGCGGAGACCGAAATGACC and 5^′^-CTCGAGTGCCTGGGAGAGCTATCTTG and cloned into the *Sma*I restriction site of pJQ200 [102] to create pCL102P. pCL102P was introduced into the genome of strain 1021 by conjugation. Transconjugants were subjected to sucrose selection on M9 agar plates with 10% sucrose, which allowed for selection of the 1021*ecfR1*^+^ strain, designated CL101. To repair *pstC*, the corresponding genomic sequence of strain RmP110 [37] was amplified using primers 5^′^-ATACTAGTTGTTCTTCTACGTTAAGAAGGCTC and 5^′^-AACTCGAGGATGAGCATGATGCCCATGACGAT and cloned into the *Spe*I and *Xho*I restriction sites of pJQ200 [102] to create pMB772. pMB772 was introduced into the genome of strain CL101 by conjugation. Transconjugants were subjected to sucrose selection on M9 agar plates with 10% sucrose, which allowed for selection of the 1021*ecfR1*^+^, *pstC*^+^ strain, designated CL150. pCAP11 is a broad host range expression vector for exogenous expression of genes from the inducible melibiose promoter, P*melA*[103]. A plasmid overexpressing *rpoE2* was constructed by amplifying the *rpoE2*-coding region from strain 1021 using primers 5^′^-GAGGATACGCGATGTCATCCGAAAACCAAG and 5^′^-AACACCTAGGTCACGAAACGAAGGCAC for 20 cycles, then adding the primer, 5^′^-GGGGCCTAGGACAGGAGGATACGCGAT, and amplifying for an additional 20 cycles. The resulting DNA was gel purified, digested with *Avr*II, and cloned into the *Avr*II site of pCAP11. Triplicate cultures of CL150 pCAP11 (vector only control) and CL150 pF1084 were inoculated to an OD_600_ of 0.05 in M9 glycerol medium and allowed to grow to midexponential phase (OD_600_=0.50-0.55), at which time melibiose was added to a final concentration of 0.4%. After 30 minutes, cells were harvested as previously described [62]. RNA purification, cDNA synthesis, labeling, hybridization to custom Affymetrix GeneChips, and data analysis were performed as previously described [62]. The custom dual-genome GeneChip contains probe sets corresponding to coding sequences from the original *S. meliloti* genome annotation reported in reference [20], intergenic regions (IGRs) of ≥ 150 nt, and to ~10,000 host plant (*Medicago truncatula*) expressed sequence tags. In designing the IGR probe sets, large IGRs were equally subdivided to be < 375 bp and tiling of oligo-(25)-mers was evenly spaced on both strands. The Affymetrix data described in this study are available in the National Center for Biotechnology Information’s Gene Expression Omnibus [104] through the GEO Series accession number GSE40391.

 5^′^ RACE (5^′^ rapid amplification of cDNA ends) was used to determine transcription start sites for a subset of *rpoE2*-dependent genes, as well as for two regulatory genes whose expression was not *rpoE2*-dependent (*rpoH1* and *rsiA2*). Primers used for 5^′^ RACE mapping are provided in Additional file 5: Table S21. Altogether, we determined transcription start sites for 26 genes, selected primarily to represent genes whose expression was detected by IGR probe sets or whose expression was previously shown to be *rpoH*-dependent [62].

## Abbreviations

bp: base pair; CDS: Coding sequence; IGR(s): Intergenic region(s); IPS: Initial promoter search; MEME: Multiple em for motif elicitation; MTU: Minimal transcription unit; ncRNA: non-coding RNA; nt: nucleotide(s); OD: Optical density; ORF(s): Open reading frame(s); Pi: Phosphate; PSSM: Position-specific scoring matrix; RACE: Random amplification of cDNA ends; RBS: Ribosome binding site; RNAP: RNA polymerase core enzyme; σ: Sigma; SD: Shine-Dalgarno; sRNA: small non-coding RNA; TSS: Transcription start site(s); UTR: Untranslated region

## Competing interests

The authors declare that they have no competing interests.

## Authors’ contributions

MJB, RG, SRL and AB designed the study. EK constructed the *expR*^+^ strain and provided the *rpoE1* data. JPS carried out the experimental work for the RNAseq approach. JPS, JR, RG and AB formulated the RNAseq data analysis strategies. JR performed the bioinformatics analyses. MJB and CL did the Affymetrix GeneChip experiments and corresponding data analysis. MJB carried out the 5^′^ RACE mapping. All authors wrote the manuscript and approved the final version. All authors read and approved the final manuscript.

## Supplementary Material

Additional file 1 Supplemental figures. Click here for file

Additional file 2: Table S1 - TSS associated with tRNAs, repeat sequences, and transposable elements; **Table S2**- TSS associated with CDS, sRNAs, and asRNA; **Table S3**- Summary of newly identified and reannotated open reading frames; **Table S4**- Comparison of experimentally validated and predicted operons to transcription start site (TSS) data; **Table S5**- Comparison of RNAseq TSS to previously published TSS. Click here for file

Additional file 5: Table S18 - Results of transcriptome analyses to identify putative RpoE2 target genes; **Table S19**- *rpoE2*-dependent genes with transcriptional activity correlated with TSS and promoter predictions; **Table S20**- *rpoE2*-dependent intergenic regions with transcriptional activity correlated with TSS and promoter predictions; **Table S21**- 5^′^ RACE mapping of TSS and comparison to RNAseq TSS; **Table S22-** Promoter-specific parameters used for promoter identification upstream of TSS; **Table S23-** Number of TSS assigned to each type of promoter sequence (virtual or model) used in the initial promoter search, by TSS category. Click here for file

Additional file 3: Table S6 - RpoD promoter sequences and their corresponding TSS; **Table S7**- RpoH1 promoter sequences and their corresponding TSS; **Table S8**- RpoH2 promoter sequences and their corresponding TSS; **Table S9**- Dual (RpoH1/H2) promoter sequences and their corresponding TSS; **Table S10**- RpoN promoter sequences and their corresponding TSS; **Table S11**- RpoE2 promoter sequences and their corresponding TSS; **Table S12**- RpoE1 promoter sequences and their corresponding TSS; **Table S13**- RpoE9 promoter sequences and their corresponding TSS. Click here for file

Additional file 4: Table S14 - Correlation of transcriptional regulator binding motifs with TSS; **Table S15**- Predicted CtrA binding motifs and their corresponding TSS; **Table S16**- Promoter motifs identified by MEME analyses; **Table S17**- Predicted FeuP binding motifs compared to TSS identified in this study and to microarray data for 14 *feuP*-dependent genes identified by Griffitts et al. [82]. Click here for file
