# Supplementary material for: Global mapping of transcription start sites and promoter motifs in the symbiotic α-proteobacterium Sinorhizobium meliloti 1021
Source: BMC Genomics. 2013 Mar 7;14:156. doi: 10.1186/1471-2164-14-156 (PMC3616915; doi:10.1186/1471-2164-14-156)
Supplement: Additional file 1 — Supplemental figures. [file 1471-2164-14-156-S1.pdf]

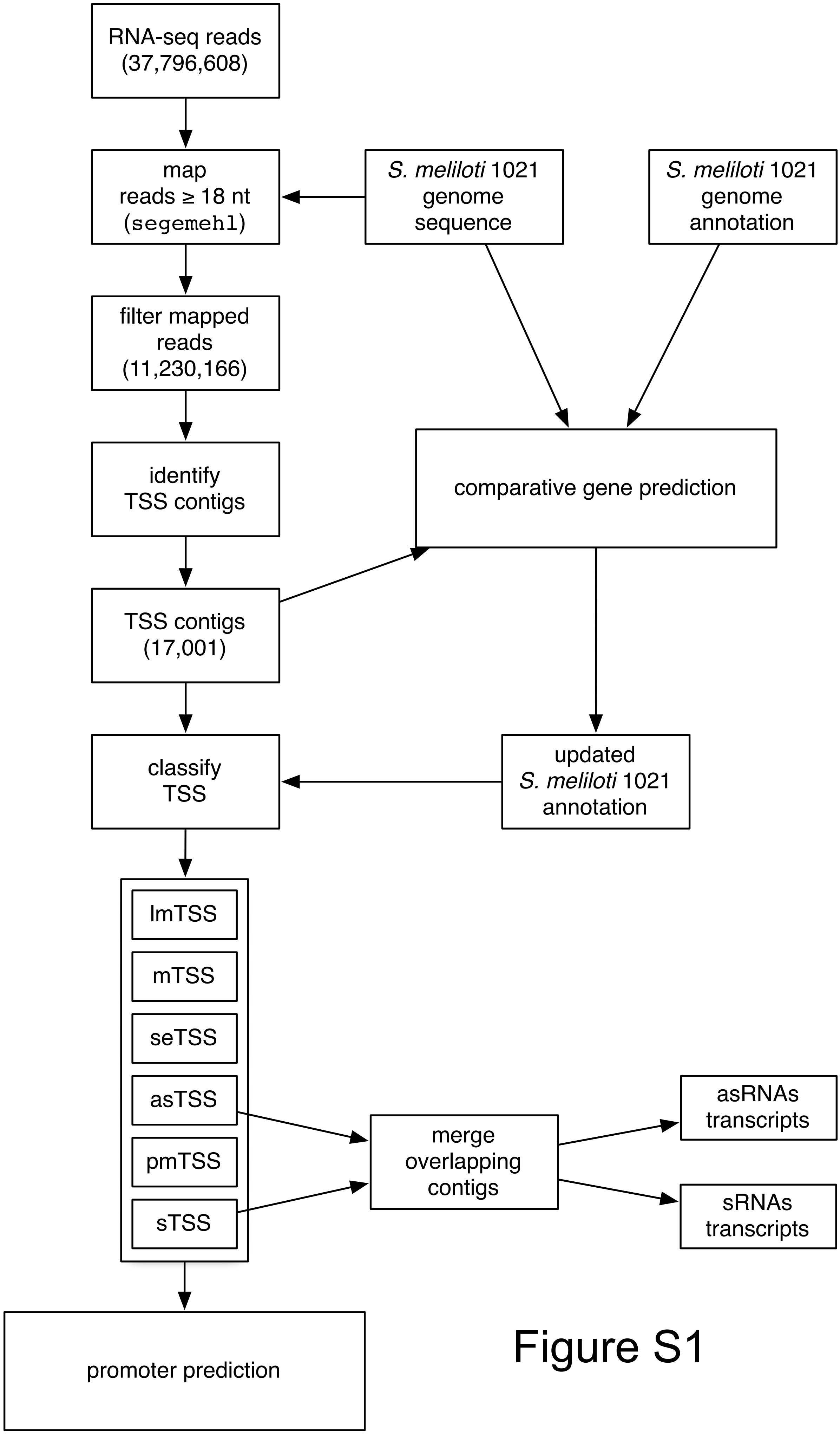
RNA-seq reads (37,796,608)
map reads ≥ 18 nt (segemehl)
S. meliloti 1021 genome sequence
S. meliloti 1021 genome annotation
filter mapped reads (11,230,166)
identify TSS contigs
comparative gene prediction
TSS contigs (17,001)
classify TSS
updated S. meliloti 1021 annotation
lmTSS
mTSS
seTSS
asTSS
pmTSS
sTSS
merge overlapping contigs
asRNAs transcripts
sRNAs transcripts
promoter prediction

Figure S1

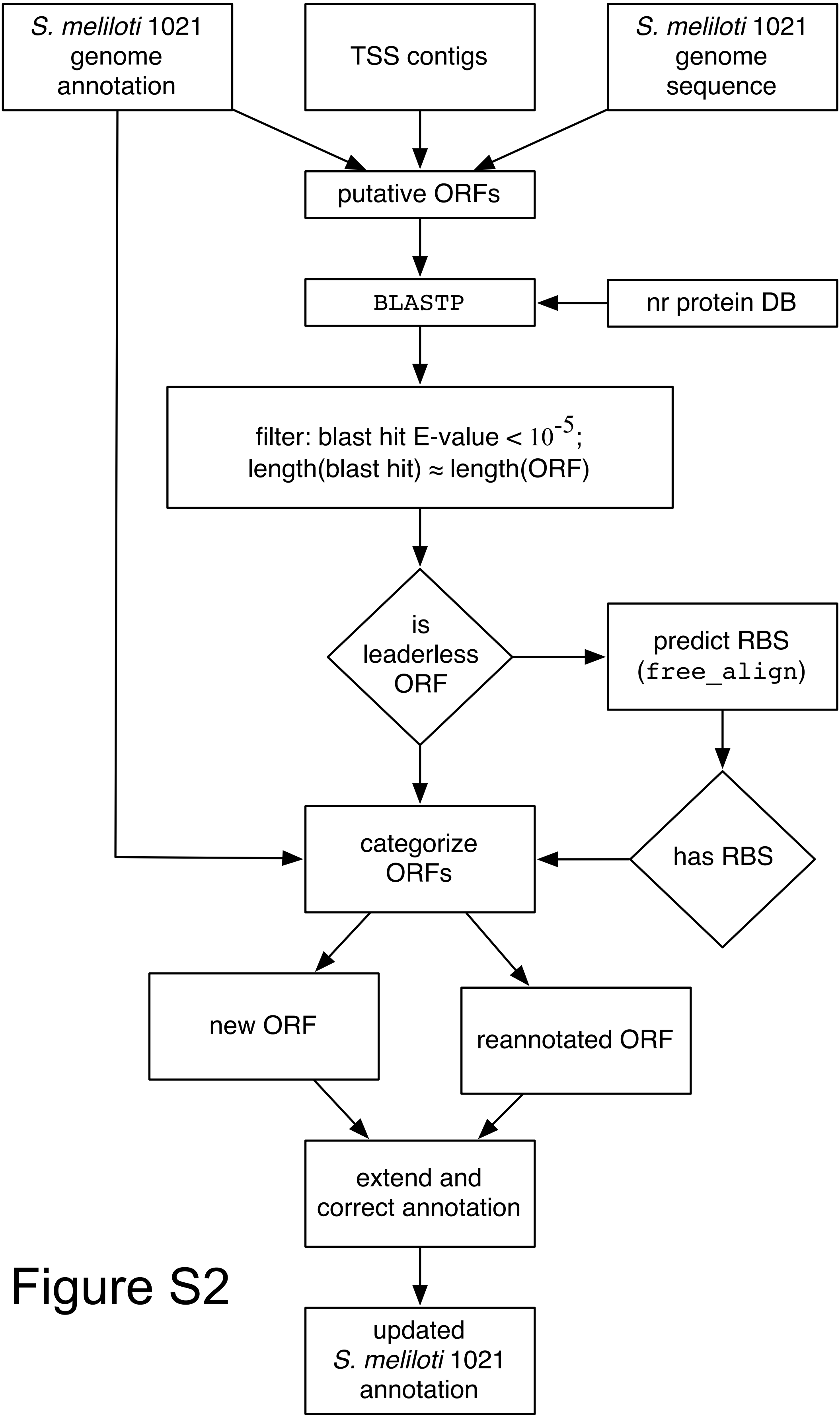

S. meliloti 1021 genome annotation
TSS contigs
S. meliloti 1021 genome sequence
putative ORFs
BLASTP
nr protein DB
filter: blast hit E-value < 10^{-5}; length(blast hit) ≈ length(ORF)
is leaderless ORF
predict RBS (free_align)
has RBS
categorize ORFs
new ORF
reannotated ORF
extend and correct annotation
updated *S. meliloti* 1021 annotation

Figure S2

Figure S3

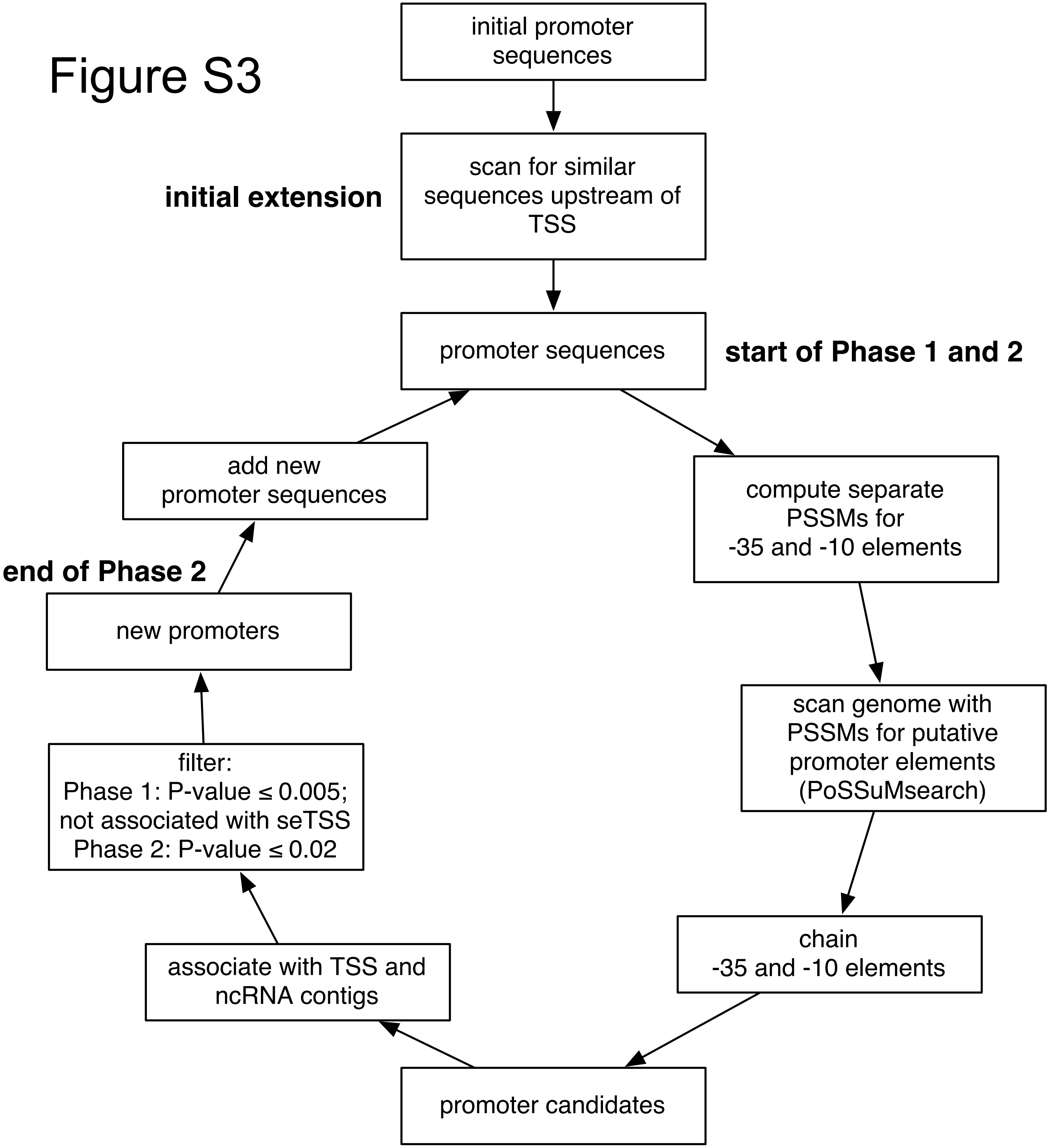
initial promoter sequences
initial extension
scan for similar sequences upstream of TSS
promoter sequences
start of Phase 1 and 2
compute separate PSSMs for -35 and -10 elements
scan genome with PSSMs for putative promoter elements (PoSSuMsearch)
chain -35 and -10 elements
promoter candidates
associate with TSS and ncRNA contigs
filter:
Phase 1: P-value ≤ 0.005; not associated with seTSS
Phase 2: P-value ≤ 0.02
new promoters
end of Phase 2
add new promoter sequences

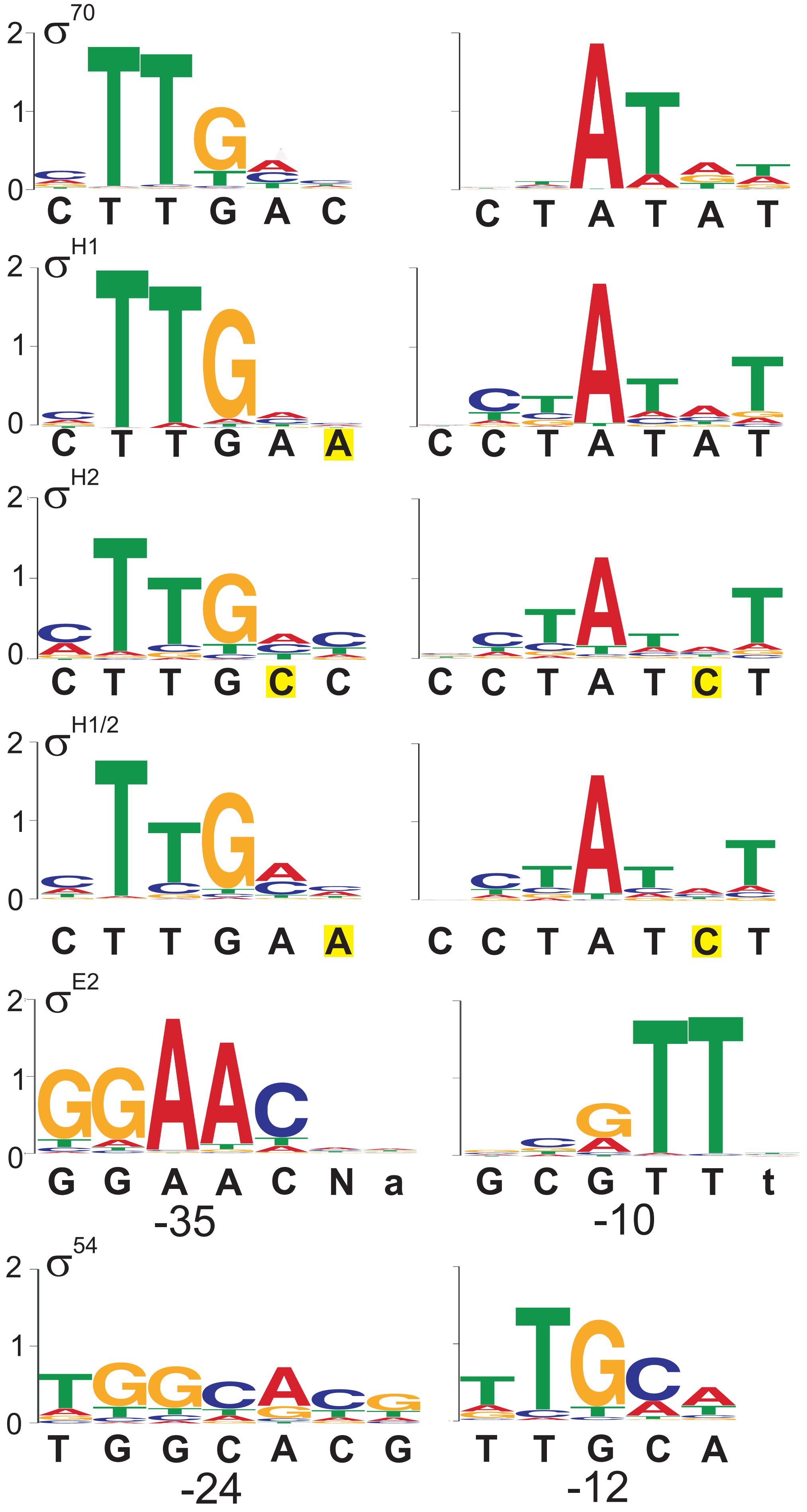

Figure S4. Consensus motifs of σ^{70}, σ^{H}, σ^{E2}, and σ^{54} promoters after addition of promoters predicted in this study. Previously published promoter consensus motifs are printed in black below the consensus motifs, with those that differ in the new consensus sequences highlighted in yellow.

Motif 1

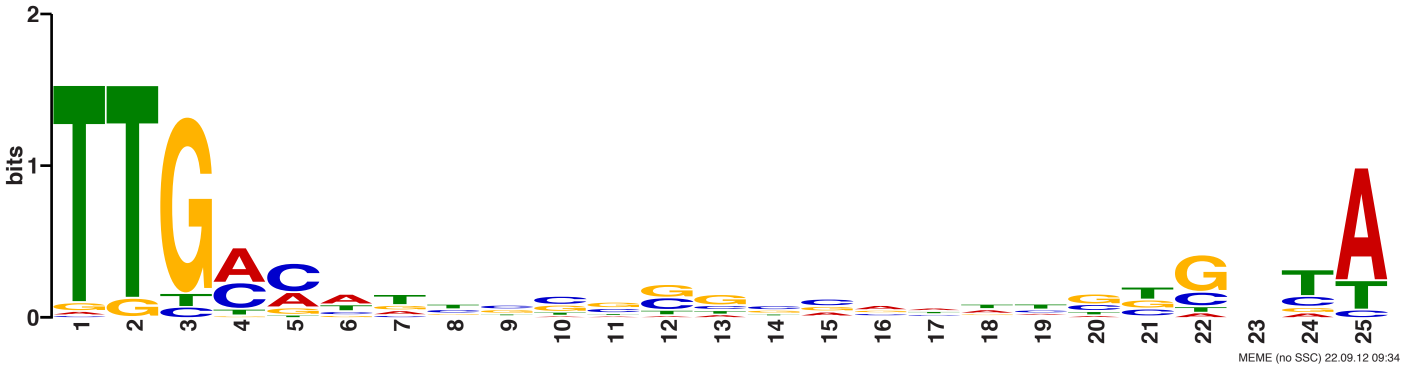

Motif 2

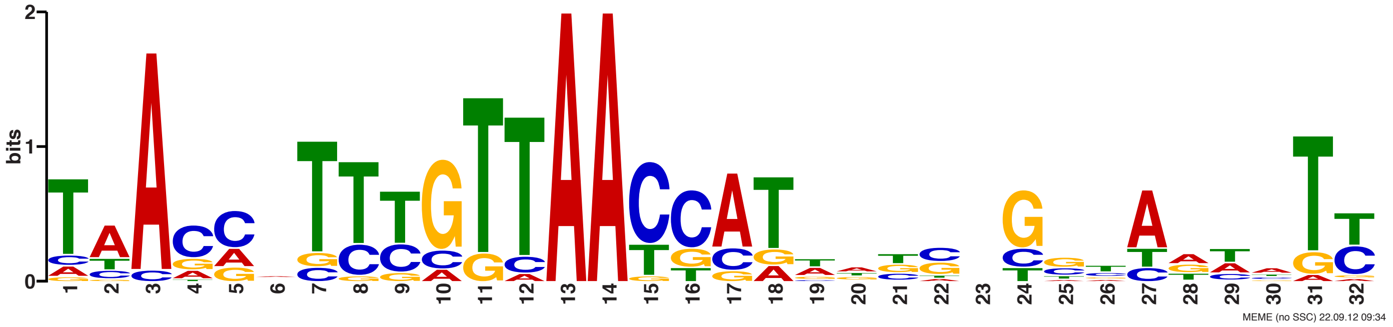

Motif 3

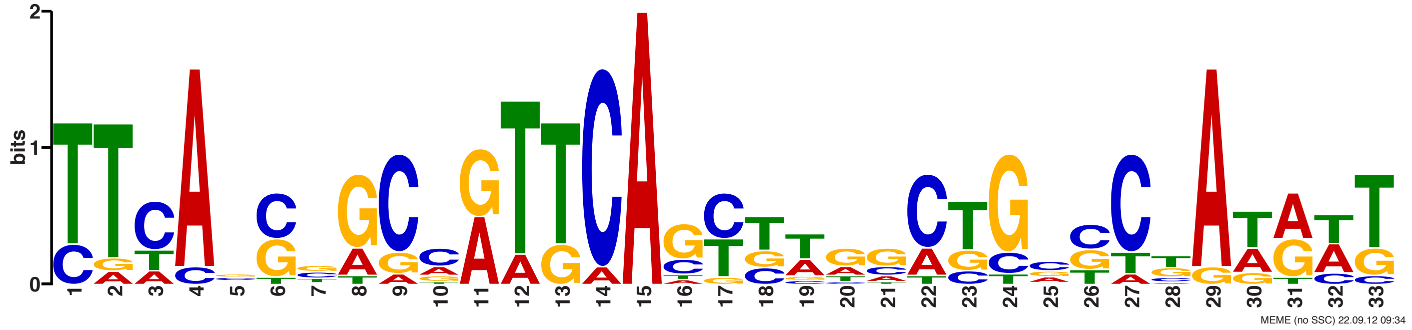

Figure S5. Motifs identified by *de novo* promoter prediction. Colored sequence logos were generated using MEME (Methods). The total information content of each logo is given in bits; the height of each nucleotide in the logo represents the positional probability of that nucleotide multiplied by the information content of the logo.

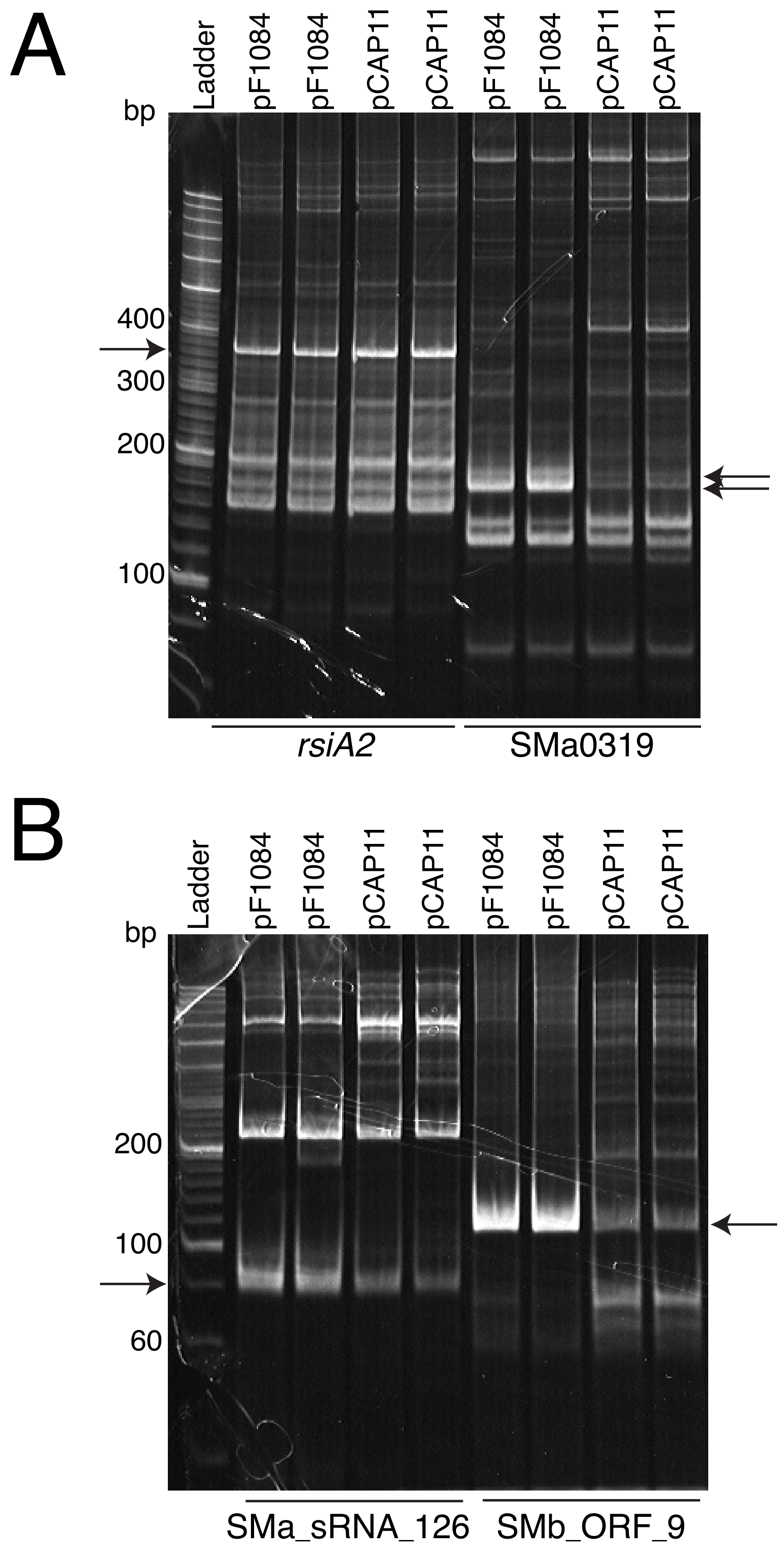

Figure S6. 5' RACE analysis of putative RpoE2 target genes. RNA from *S. meliloti* CL150 cells overexpressing *rpoE2* (pF1084) or carrying an empty vector (pCAP11) was ligated to an RNA primer and reverse transcribed to produce cDNA for 5' RACE. PCR products were resolved by polyacrylamide gel electrophoresis and visualized by SYBR Gold staining. Representative gels for two protein coding genes are shown in A). *rsiA2* (SMc04884) expression was not *rpoE2*-dependent in our Affymetrix experiments, whereas expression of SMa0319 was *rpoE2*-dependent. Gels for genes newly identified in this study are shown in B). Expression of a non-coding RNA (SMa_sRNA_126), and an ORF (SMb_ORF_9), is *rpoE2*-dependent. Bands that were excised and sequenced for transcription start site determination are indicated by an arrow.
